# Injectable and In Situ Hydration‐Reinforced Hybrid Bone Cements for Accelerated Bone Regeneration

**DOI:** 10.1002/advs.202512723

**Published:** 2025-11-16

**Authors:** Xing Chen, Yifan Ma, Lingfei Zhao, Lingbin Che, Dianwen Song, Zihan Wu, Changsheng Liu, Yuan Yuan

**Affiliations:** ^1^ Key Laboratory for Ultrafine Materials of Ministry of Education School of Materials Science and Engineering East China University of Science and Technology Shanghai 200237 P. R. China; ^2^ Engineering Research Center for Biomedical Materials of the Ministry of Education East China University of Science and Technology Shanghai 200237 P. R. China; ^3^ Department of Radiation Oncology The University of Texas MD Anderson Cancer Center Houston TX 77030 USA; ^4^ Department of Orthopedics Shanghai General Hospital Shanghai Jiaotong University School of Medicine Shanghai 200080 P. R. China; ^5^ Shanghai Rebone Biomaterials Co., Ltd Shanghai 201707 P. R. China

**Keywords:** bone regeneration, hydration reinforcement, injectable hybrid bone cement, in situ pore formation, linear polyhydroxy PEGylated poly(glycerol sebacate) (L‐PEGS)

## Abstract

Injectable bone cements are recognized as ideal solution for bone augmentation due to their minimally invasive introduction strategy. However, current clinical formulations and composites, while demonstrating improvements in certain areas, often fail to comprehensively address challenges, including rheological injectability, mechanical stability, interconnected porosity, degradability, and bioactivity. To overcome limitations, a novel bone cement comprising linear polyhydroxy PEGylated poly(glycerol sebacate) (L‐PEGS) and calcium phosphate cement (CPC) is developed. The incorporation of L‐PEGS enhances injectability and reinforces the mechanical weakness of CPC, resulting in improvement in compressive strength (increased by 16.7 folds) and fatigue resistance (1000 cycles). Owing to linear polyhydroxy backbone, L‐PEGS initiates self‐reinforcing cross‐linking reaction synchronized with the hydration of CPC into hydroxyapatite. This hydration reinforcement is mediated by its abundant hydroxyl groups and high water absorption capacity, which accelerate hydration kinetics. Concurrently, the cross‐linking reaction generates in situ carbon dioxide, resulting in porous microarchitecture that facilitates hydration process, enhances cement degradability, and promotes nutrient exchange and new bone ingrowth. In vitro and vivo studies confirmed that L‐PEGS/CPC substantially enhances osteogenesis compared to clinical materials. Collectively, this injectable, hydration‐driven, and self‐reinforcing bone cement offers comprehensive solution to the challenges in current bone graft materials, holding promise for clinical bone repair.

## Graphical abstract

To enable minimally invasive bone defect repair, an injectable and hydration‐reinforced bone cement (L‐PEGS/CPC) is designed through biomimetic reconstruction. The hydrophilic L‐PEGS organic phase provides abundant nucleation sites, synergizing with its porous architecture to accelerate CPC hydration, thereby endowing the composite with exceptional mechanical strength and fatigue resistance—offering a novel strategy for clinical bone repair materials.

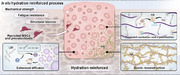

## 1 Introduction

Complex bone tissue defects, arising from diseases, trauma, or congenital abnormalities, are increasingly prevalent clinical challenges driven by an aging population, which in turn fuels the urgent need for effective bone substitutes.^[^ ^1^, ^2^, ^3^, ^4^ ^]^ While commercial bone cements, mainly poly (methyl methacrylate) (PMMA), are widely used, their clinical performance is compromised by several drawbacks, including poor degradability, exothermic curing process, high modulus that creates mechanical mismatches with native tissues, and biological inertness.^[^ ^5^, ^6^, ^7^ ^]^ In response, calcium phosphate (CaP)‐based biomaterials, particularly calcium phosphate cement (CPC), has garnered attention due to their biomimetic composition, excellent biocompatibility, degradability, and availability in various forms, such as allogenic, sintered, or injectable cementitious materials.^[^ ^8^, ^9^, ^10^, ^11^, ^12^ ^]^ Unfortunately, CaP‐based bone cements also suffer from critical limitations, most notably poor mechanical stability and intrinsic brittleness. These deficiencies complicate surgical manipulation and compromise post‐implantation stability, as the cement's inability to maintain its integrity under physiological conditions leads to washout by body fluids, forming large granules or fragments that provoke inflammatory responses.^[^ ^13^, ^14^, ^15^ ^]^ Moreover, limited degradability, along with the absence of sufficient porosity and pore interconnectivity hinders the effective transfer of nutrients and cellular components essential for the ingrowth of new bone.^[^ ^16^, ^17^, ^18^ ^]^ Thus, numerous efforts have been directed toward addressing these challenges for clinical bone augmentation.

To better meet clinical requirements, various approaches have been employed to modify and enhance the performance of CPC. Adjusting the powder‐to‐liquid ratio^[^ ^19^ ^]^ and reducing the initial particle size^[^ ^20^ ^]^ can improve the compressive strength of CPC; however, such modifications may simultaneously affect key physicochemical properties—such as porosity, washout resistance, and degradability—potentially triggering inflammatory responses and impairing bone regeneration.^[^ ^21^ ^]^ To further improve the biological activity of CPC, trace elements such as strontium (Sr), zinc (Zn), and copper (Cu)—which play physiological roles in bone anabolism—have been incorporated.^[^ ^22^, ^23^ ^]^ Nevertheless, these additions can also alter the dissolution behavior of CPC, resulting in a retardation of material degradation rates.^[^ ^24^ ^]^ Alternatively, the introduction of polymeric phases into CPC formulation have been widely used to enhance the cohesion and mechanical properties of the bone cement. For instance, synthetic polymers such as poly(lactic‐co‐glycolic acid) (PLGA) and poly(ε‐caprolactone) (PCL) have been utilized to enhance CPC's cohesive strength and address inherent brittleness and structural instability.^[^ ^25^, ^26^ ^]^ However, these hydrophobic polymers are typically water‐insoluble and require pre‐processing via oil‐phase blending or powder‐phase composite formation.^[^ ^27^, ^28^ ^]^ Such techniques like granulation, paste mixing or post‐coating often lead to phase separation, impairing mechanical stability, disrupting porous interconnectivity, ultimately hindering in vivo bone mineralization and vascularization. Consequently, these existing modifications address only a subset of CPC's limitations and often introduce new challenges. Therefore, it is imperative to develop a facile and efficient strategy based on a specific formulation that achieves an optimal balance among injectability, mechanical stability, interconnected porosity, and degradability, ultimately overcoming the inherent limitations of conventional CPC and other commercial bone cements.

CPC is considered a biomimetic scaffold not only due to its chemical composition resembling natural bone, but also because of its ability to transform into bone‐like mineral phases under physiological conditions through a hydration‐driven process.^[^ ^29^ ^]^ Specifically, the hydration process of CPC bone cement begins with the dissolution of surface calcium phosphate salts, releasing calcium ions (Ca^2^⁺) and phosphate ions (PO_4_^3−^). Subsequently, as the ion concentration increases, the solution reaches a supersaturated state, triggering the nucleation and crystallization of CPC into hydroxyapatite (HA) crystals. Finally, water molecules penetrate through the surface HA layer, further promoting the formation of an interconnected HA network within the CPC. As the hydration process progresses, CPC gradually transforms into HA, resulting in enhanced mechanical strength and improved osteoconductivity, ultimately promoting robust osseointegration at the defect site. The kinetics and efficiency of this hydration process are primarily influenced by factors such as CPC particle size, specific surface area, and humidity.^[^ ^30^, ^31^, ^32^ ^]^ In particular, the rate of nucleation and crystal growth during early‐stage hydration is governed by the surface dissolution of the precursor materials. A higher specific surface area not only accelerates ion release and HA formation, but also facilitates the diffusion of water molecules into the interior of the cement.^[^ ^33^ ^]^ Similarly, elevated humidity levels enhance the dissolution of powder surfaces and promote spontaneous water penetration, thereby expediting the overall hydration reaction. Inspired by these underlying hydration mechanisms, we propose a novel composite formulation designed to facilitate and optimize the hydration process, thereby comprehensively enhancing the performance of CPC.

**Scheme 1:**
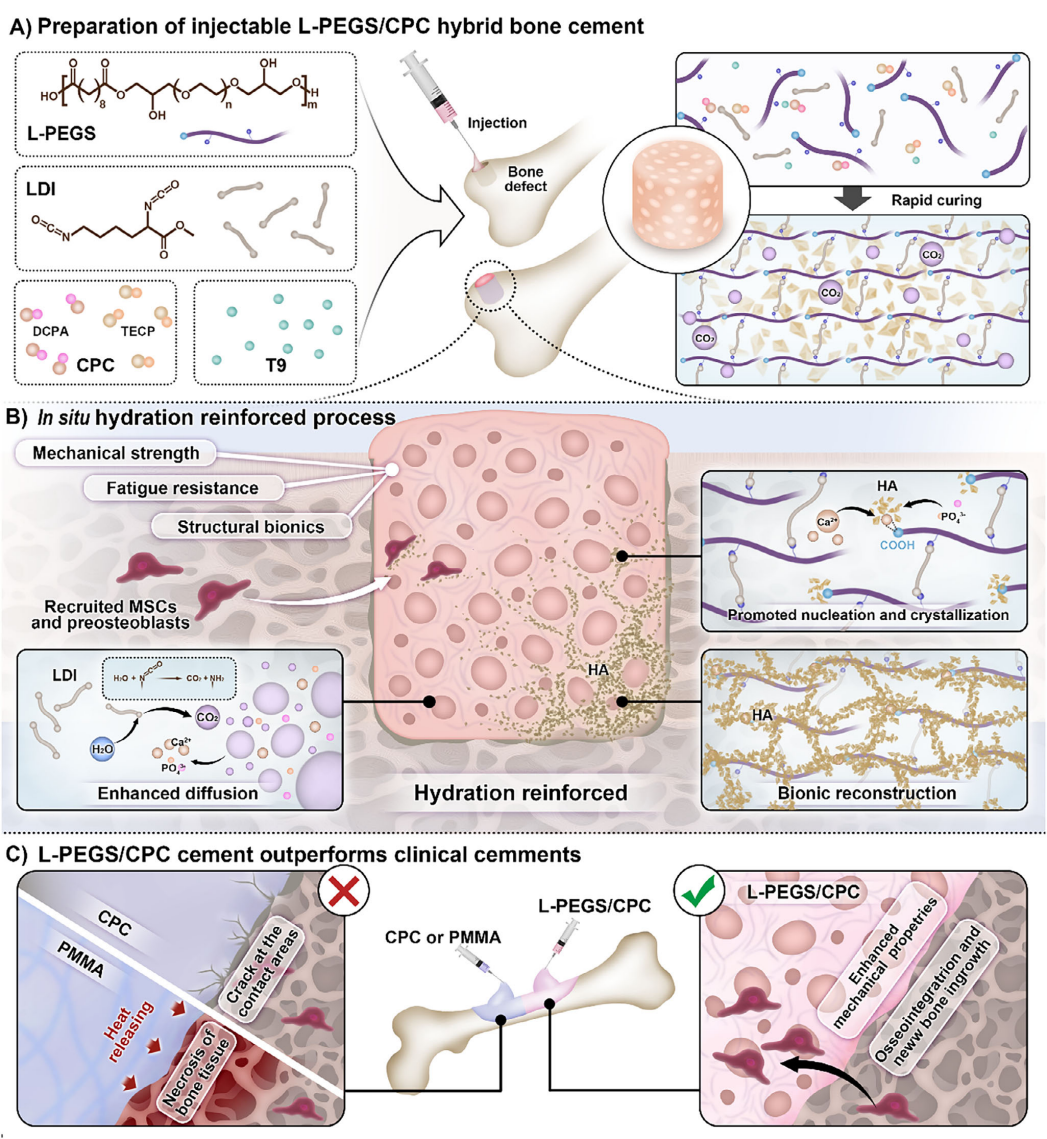
Schematic of the injectable, in situ hydration‐reinforced L‐PEGS/CPC hybrid bone cement designed to enhance bone regeneration. A) The L‐PEGS/CPC composite, composed of L‐PEGS precursors, initiator and catalyst (LDI and T‐9), and CPC precursors (DCPA and TECP), exhibits excellent injectability and undergoes rapid in situ solidification within 15 min after injection into bone defects. B) First, environmental water can react with LDI, generating an interconnected porous architecture. The synergistic interplay between the porous architecture and hydrophilic L‐PEGS accelerates the dissolution of calcium and phosphate ions through rapid aqueous permeation. Second, the carboxyl groups on L‐PEGS coordinate with liberated calcium ions, thereby enhancing both the nucleation and crystalline of HA during biomimetic mineralization. Finally, the chemically cross‐linked organic network orchestrates oriented HA crystallization, enabling biomimetic reconstruction that yields an enhancement in load‐bearing capacity. C) The L‐PEGS/CPC composite exhibits excellent injectability, superior mechanical strength, and enhanced bioactivity, addressing the mechanical fragility, excessive exothermic reaction, and poor biointegration associated with the bioinert nature of clinically available bone cements.

While the incorporation of polymers into CPC can effectively address its poor mechanical properties, phase separation often arises due to the high solubility of many polymers in oil phases, thereby interfering with CPC hydration. In contrast, polymers with high aqueous solubility and autonomous cross‐linking capabilities can effectively mitigate phase segregation while forming an in situ cross‐linked network that enhances the cohesive strength of the cement. Furthermore, their hydrophilic nature and functional group composition are supposed to synergistically accelerate CPC hydration kinetics, facilitating faster in vivo HA transformation. Among various candidates, PEGylated poly (glycerol sebacate) (PEGS)—a biodegradable elastomer derived from poly(glycerol sebacate)—has attracted significant interest due to its favorable physical and mechanical properties, as well as versatility in processing and molding.^[^ ^34^, ^35^, ^36^, ^37^, ^38^ ^]^ As a water‐soluble polymer, PEGS can integrate with the CPC hydration process to form a cohesive organic–inorganic hybrid network. Its hydroxyl‐rich backbone enhances hydrophilicity, thereby promoting ion exchange and hydration, while its linear molecular architecture improves injectability and flow behavior. In this study, we developed an injectable, hydration‐reinforced, porous‐forming composite scaffold composed of linear polyhydroxy PEGylated poly (glycerol sebacate) (L‐PEGS) and CPC, aiming to enhance bone regeneration. L‐PEGS was synthesized via acid‐induced epoxy ring‐opening polymerization, yielding a more uniform molecular structure, improved solubility, and higher hydroxyl group density compared to conventional branched PEGS (B‐PEGS). As depicted in the **Scheme** **1**, the cement forms an in situ organic–inorganic interpenetrating network, where L‐PEGS facilitates a self‐reinforcing cross‐linking reaction that occurs synchronously with CPC hydration and mineralization. Specifically, L‐lysine diisocyanate (LDI) cross‐linked PEGS initiates polymerization, enabling cross‐linking and curing of the bone cement through a nucleophilic addition reaction, forming a cross‐linked polymeric matrix. During this process, water undergoes nucleophilic addition with LDI, releasing carbon dioxide, which promotes the development of interconnected pores within the composite cement. This porous architecture increases the specific surface area, thereby accelerating the hydration process and facilitating nutrient exchange, cell migration, and tissue ingrowth—key factors for effective bone regeneration. At the same time, the high hydrophilicity of L‐PEGS, attributed to its abundant hydroxyl groups, works in concert with the increased surface area to further enhance CPC hydration, ultimately improving the mechanical properties of the cement. This synchronous organic–inorganic network is believed to endow the scaffold with superior mechanical strength, resistance to dispersion, degradability, and favorable cell responses and bioactivity, collectively promoting osteogenesis. This L‐PEGS/CPC composite cement was further evaluated in a femoral defect model using Sprague‐Dawley (SD) rats, with a comparison to clinically used PMMA and CPC, to assess its bone regeneration capacity and clinical translation potential.

## 2 Results and Discussion

### 2.1 Synthesis and Characterization of Branched‐PEGS (B‐PEGS) and Linear‐PEGS (L‐PEGS)

**Figure 1:**
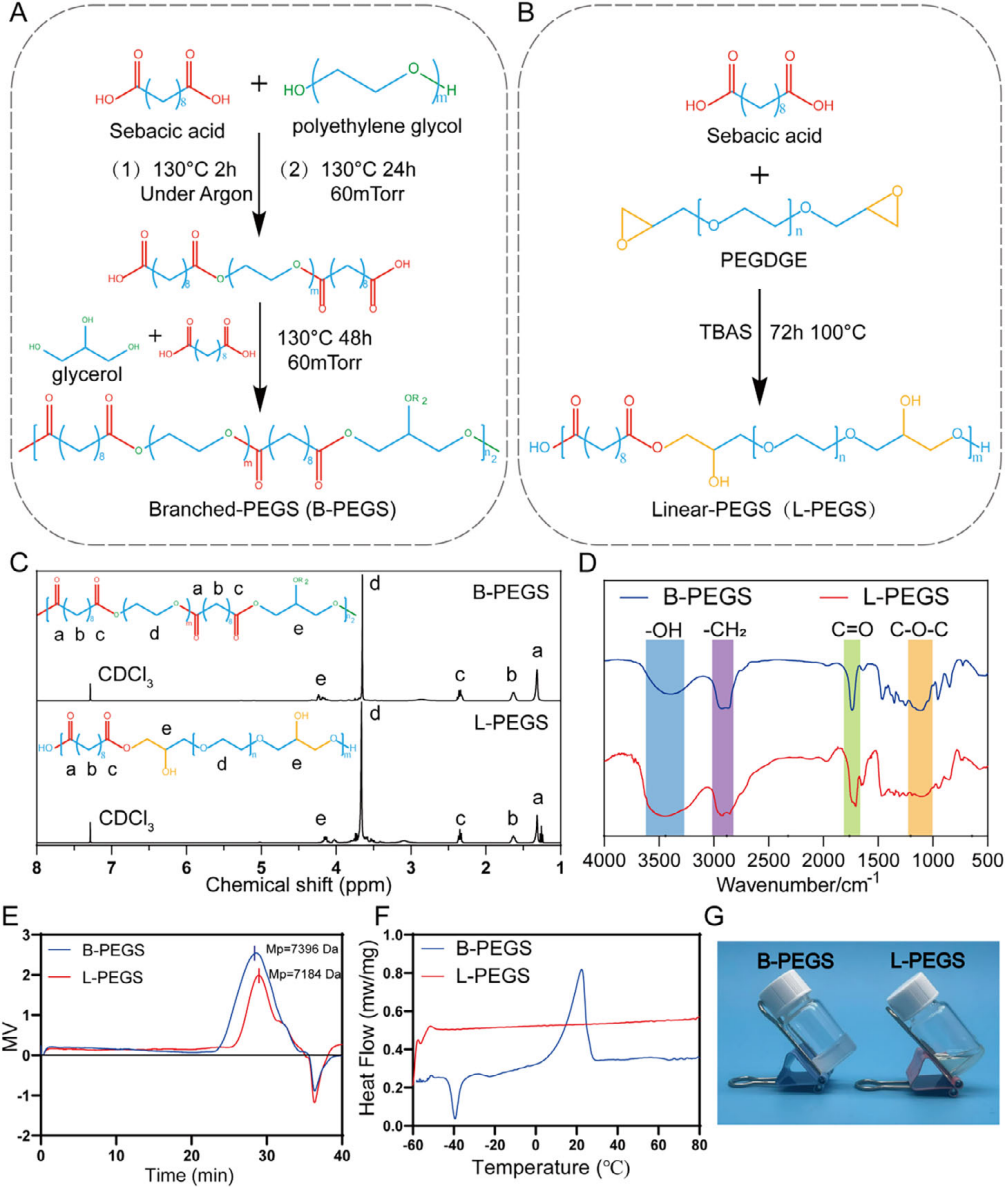
Synthesis and characterization of B‐PEGS and L‐PEGS. Synthetic schemes of A) B‐PEGS and B) L‐PEGS. C) ^1^H NMR spectra of B‐PEGS and L‐PEGS. Chemical shift peaks of sebacic acid, polyethylene glycol and glycerol were observed at 1.2, 1.54, 2.37, 3.62, and 4.18 ppm, respectively for B‐PEGS and L‐PEGS, indicating the successful synthesis of PEGS with different structures. D) FT‐IR spectrum of L‐PEGS and B‐PEGS in the range of 500–4000 cm^−1^. The hydroxyl peak (3400 cm^−1^) of L‐PEGS was stronger than that of B‐PEGS, indicating more hydroxyl content. E) GPC curves of L‐PEGS and B‐PEGS. At comparable molecular weight level, L‐PEGS exhibits a narrower molecular weight distribution, indicating higher homogeneity. F) DSC curves of L‐PEGS and B‐PEGS. G) L‐PEGS remains in a fluidic state, whereas B‐PEGS exhibits a creamy, colloidal‐like state.

To address the inherent issues of CPC through the incorporation of polymers, water‐soluble PEGS was synthesized. Given that the structure of the polymer may potentially influence the reinforcement of material properties, particularly its impact on the hydration process, we initially synthesized PEGS with distinct molecular chain architectures using two different synthetic approaches. Specifically, B‐PEGS was synthesized via a two‐step condensation polymerization process (**Figure** **1**A). The first step involves the condensation polymerization of sebacic acid and polyethylene glycol (Mn = 1000 Da) under vacuum conditions, followed by the second step, in which glycerol is introduced to undergo further condensation polymerization with sebacic acid and the pre‐polymer formed in the first step. In contrast, L‐PEGS was synthesized via TBAS‐induced epoxy ring‐opening reaction (Figure 1B). The acid‐induced epoxide ring‐opening reaction provides a more streamlined synthetic approach with milder reaction conditions compared to the two‐step condensation polymerization process. In comparison to B‐PEGS, L‐PEGS is supposed to exhibit a higher density of hydroxyl groups along its molecular chains due to the nucleophilic attack of carboxylate anions on epoxide rings. Variations in the phase of the polymer reinforcement can affect both its intrinsic performance and the post‐injection hydration process, which ultimately contribute to strengthening the CPC composite, leading to an overall enhancement of its properties.

The successful synthesis of B‐PEGS and L‐PEGS were characterized using Nuclear Magnetic Resonance (NMR) spectroscopy and Fourier Transform Infrared (FTIR) spectroscopy. The stretching vibrational peaks corresponding to hydroxyl (–OH), methylene (–CH_2_–), carbonyl (─C═O), and ether (–C–O–C–) bonds within the structure of B‐PEGS and L‐PEGS appeared at wavenumbers of 3400, 2950, 1750, and 1100 cm^−1^, respectively, as analyzed by FT‐IR spectroscopy (Figure 1D). Comparative analysis reveals that the hydroxyl absorption peak (λ = 3500 cm^−1^) of L‐PEGS is significantly stronger than that of B‐PEGS. As shown in Figure 1C, the methylene groups (a, b, c) in the three distinct chemical environments of sebacic acid within B‐PEGS and L‐PEGS exhibit corresponding peaks at 1.20, 1.54, and 2.37 ppm, respectively. Furthermore, the methylene proton peak (d) connected to the oxygen atom in both B‐PEGS and L‐PEGS appeared at 3.62 ppm, and the hydroxyl peak is also included in it. Meanwhile, the methylene peaks (e) in glycerol of both B‐PEGS and L‐PEGS exhibit a resonance peak at 4.18 ppm. Notably, the absence of the epoxy‐based proton signal (δ∼3.00 ppm) indicates complete depletion of the epoxy functional group for the synthesis of the L‐PEGS. Through acid‐base titration experiments, the hydroxyl content of L‐PEGS was increased by 4 times compared to B‐PEGS (Table S1, Supporting Information). The increased hydroxyl content in L‐PEGS provides a greater number of active sites, resulting in significantly higher reactivity compared to B‐PEGS, while also endowing L‐PEGS with enhanced hydrophilicity.

After the confirmation of the successful synthesis of two different PEGS bases, we then identify the structure and morphology differences. The polydispersity index (PDI) serves as a critical metric for evaluating the uniformity of molecular weight distribution, and through optimization of the synthetic process, an ideal molecular weight distribution can be achieved. As determined by gel permeation chromatography (GPC), the weight‐average molecular weights (Mw) of B‐PEGS and L‐PEGS were measured to be 11,970 and 10,633 Da, respectively. Notably, L‐PEGS exhibited a significantly narrower PDI of 1.30 compared to 3.74 for B‐PEGS, indicating a more uniform performance profile for L‐PEGS (Figure 1E; Figure S1E and Table S1, Supporting Information). The crystallinity of polymeric materials plays a pivotal role in determining their processability and composite‐forming characteristics with CPC. Therefore, the crystallinity of B‐PEGS and L‐PEGS was systematically investigated using differential scanning calorimetry (DSC). B‐PEGS exhibits a glass transition temperature (T_g_) of −51.3 °C, a melting temperature (T_m_) of 22.4 °C, and a crystallization temperature (T_c_) of −39.5 °C (Figure 1F; and Table S1, Supporting Information). L‐PEGS, in contrast, exhibits no detectable T_g_, T_m_, or T_c_ within the temperature range of −60 °C to 80 °C, indicating a significantly broader operational temperature range compared to B‐PEGS. At room temperature, L‐PEGS exhibits a fluid‐like state, whereas B‐PEGS demonstrates a paste‐like consistency (Figure 1G). Owing to its superior fluidity, L‐PEGS enables rapid and homogeneous mixing with CPC powder, significantly reducing the pre‐processing time required for material preparation. The higher crystallinity of B‐PEGS compared to L‐PEGS leads to a greater tendency for phase separation when blended with CPC, requiring significantly higher mechanical force to achieve a homogeneous distribution of the two phases.

### 2.2 Preparation and Formulation Optimization of PEGS/CPC Bone Cement

**Figure 2:**
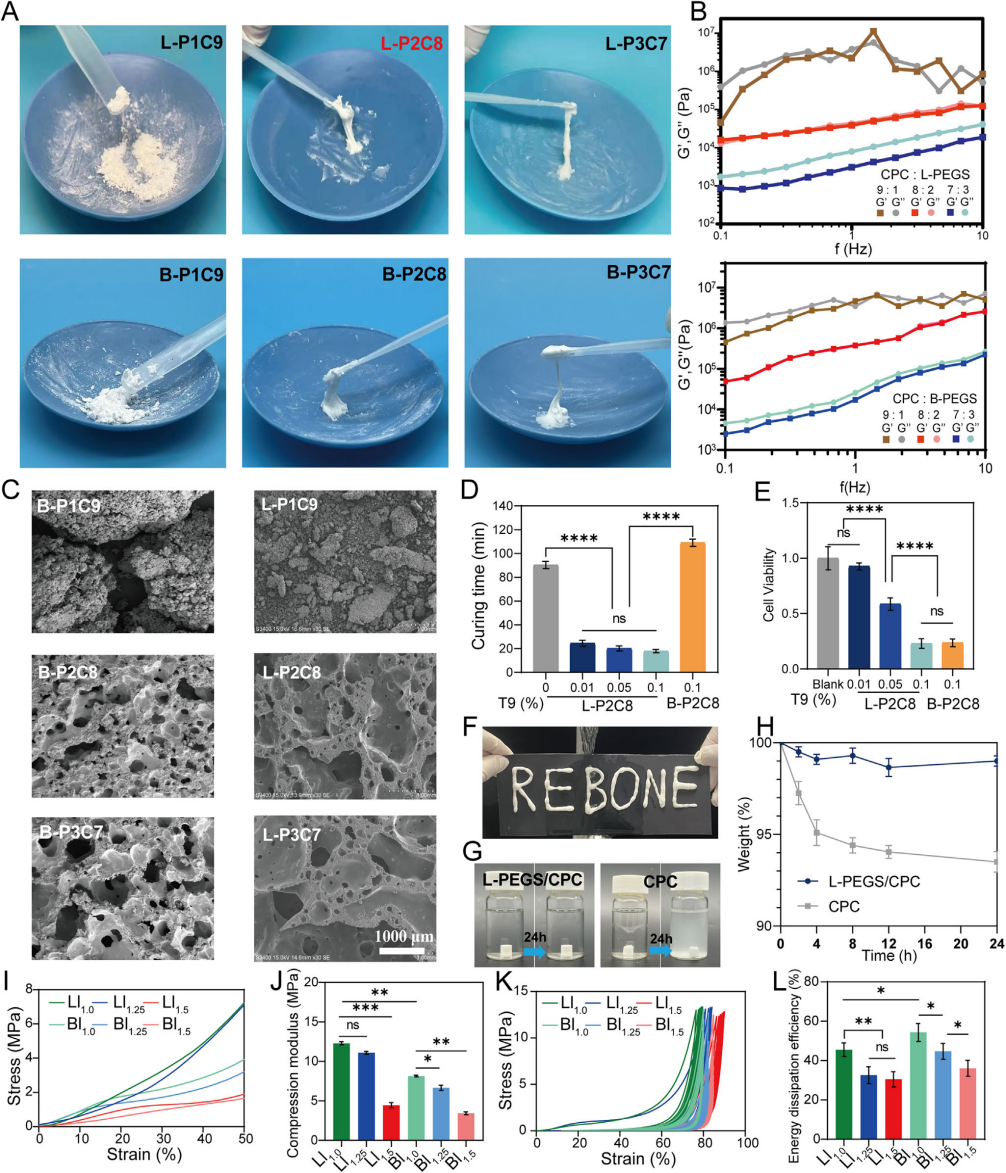
Preparation and formulation optimization of PEGS/CPC bone cement. A) State of bone cement slurries with different PEGS/CPC composite ratios (denoted as L or B‐PxCy, where x: y represents the PEGS: CPC mass ratio). Increasing PEGS content enhances the flowability of the slurry. At a PEGS: CPC ratio of 2:8, the slurry retains sufficient fluidity while exhibiting high viscosity, effectively minimizing the risk of dispersion into surrounding tissue. B) Flowability assessment of different PEGS/CPC formulations via rotational rheometry. Compared to B‐PEGS/CPC, L‐PEGS/CPC has no significant shear thickening and shows better injectability. C) SEM images of bone cement with various PEGS/CPC ratios. (Scale bar: 1000 µm) D) The curing time of L‐PEGS/CPC and B‐PEGS/CPC with varying amounts of stannous octanoate catalyst. E) The cytotoxicity of L‐PEGS/CPC and B‐PEGS/CPC with varying amounts of stannous octanoate catalyst toward L929 fibroblasts. (Materials must first satisfy minimum cytotoxicity thresholds stipulated by national standards to demonstrate translational potential, thereby qualifying for subsequent cell‐specific investigations.) F) L‐PEGS/CPC maintains excellent resistance to water washout. G) The anti‐dispersive properties of L‐PEGS/CPC and CPC after solidification. H) The residual mass ratio of L‐PEGS/CPC and CPC after continuous soaking, indicating that the L‐PEGS network effectively overcomes CPC's vulnerability to collapse during the pre‐curing phase. I) Compressive stress–strain curves and J) modulus of bone cement with various LDI amount. K) Cyclic loading‐unloading compressive curves and L) energy dissipation efficiency over 10 cycles for bone cements with various LDI amount at a compressive distance of 4 mm. (Data are represented as mean ± SD; *n* = 5; *p* ≥0.05 (no significant, ns), *p < 0.05; **p < 0.01; ***p < 0.001, and ****p < 0.0001).

Undesirable or delayed solidification of bone cement paste following injection increases the risk of leakage, which can lead to serious complications such as nerve compression and vascular occlusion.^[^ ^39^, ^40^ ^]^ To prevent post injection leakage of bone cement into the bone defect site in vivo, it is crucial for the cement to solidify rapidly and reduce its flowability. To balance injectability and rapid setting, the composite ratio of PEGS (liquid phase) to CPC (solid phase) was systematically optimized. The resulting composite paste, designated as L or B‐PxCy—where L and B refer to linear PEGS (L‐PEGS) and branched PEGS (B‐PEGS), and x and y denote the mass fractions of PEGS and CPC, respectively—was evaluated for flow behavior across various formulations. Thermogravimetric analysis (TGA) was first used to confirm the organic‐to‐inorganic ratio within the composite. As shown in the Figure S1G (Supporting Information), PEGS began decomposing at approximately 250 °C, whereas CPC remained thermally stable up to 800 °C. The residual masses of P1C9, P2C8, and P3C7 were 88.77%, 79.12%, and 70.11%, respectively, indicating consistency between the theoretical and experimental feeding ratios. After confirming the exact formulation of the composite, injectability of the mixture was assessed. When the ration of PEGS to CPC exceeded 5:5, the composite paste demonstrated favorable flowability and low viscosity. With increasing CPC content, however, the paste's consistency shifted toward a toothpaste‐like texture (Figure S1A, Supporting Information). Notably, the L‐P2C8 formulation exhibited excellent injectability with controlled flow, achieving an optimal toothpaste‐like consistency (**Figure** **2**A). While the bone cement with the B‐P2C8 ratio also exhibited a toothpaste‐like consistency, its flowability was inferior to that of the L‐P2C8 formulation, attributed to the inherently higher viscosity of B‐PEGS at room temperature. In contrast, formulations with higher CPC content, such as L‐P1C9 and B‐P1C9, formed a flour‐like texture lacking injectability (Figure 2A), due to the reduced liquid phase and increased solid content.

After initial screening, the rheological properties of bone cement pastes with six different ratios (L or B‐P1C9, L or B‐P2C8, and L or B‐P3C7) were selected and further analyzed (Figure 2B). As the inorganic CPC component increased, both the storage modulus (G′) and loss modulus (G″) also increased. For the L‐P3C7 and B‐P3C7 bone cement paste, the G″ value surpassed G′, indicating a more liquid‐like state with noticeable fluidity. The L‐P2C8 and B‐P2C8 paste displayed G″ and G′ values in close proximity, indicating an intermediate state between solid and liquid, which allowed for controlled injectability and limited flow. Finally, the L‐P1C9 and B‐P1C9 bone cement paste, resembling a solid powder‐like state, dissipated external energy through particle sliding, resulting in a rheological profile where G″ exceeded G′. In addition, the B‐PEGS/CPC bone cement, due to the higher degree of branching in B‐PEGS, is prone to molecular chain entanglement, resulting in shear thickening behavior, which leads to lower injectability compared to the L‐PEGS/CPC formulation. The rheological behavior and injectability may be further modulated by polymeric zeta potential. As shown in Figure S1D (Supporting Information), L‐PEGS exhibited 8.22‐fold greater absolute zeta potential values than B‐PEGS, with a pronounced positive surface charge. This elevated surface charge density induces interparticle electrostatic repulsion within the liquid phase, enhancing cement particle dispersion and ultimately facilitating injectability.^[^ ^41^, ^42^ ^]^ Therefore, P2C8 can maintain fluidity during injection or be molded into any shape after injection. Based on the aforementioned results, we can conclude that a solid‐to‐liquid ratio of 8:2 is the optimal formulation for achieving desirable flowability and injectability in bone cement. Accordingly, the P2C8 bone cement not only ensured excellent injectability but also exhibited high viscosity, effectively minimizing the risk of undesired cement diffusion within the defects. This formulation was therefore selected for subsequent experiments.

Following the optimization of the injectability of the material, the setting properties were further tuned by adjusting the content of cross‐linking agents and catalysts. Beyond the component formulation in PEGS/CPC, the roles of LDI and catalyst T9 are crucial for the in situ pore formation and curing process of the cement. Specifically, the isocyanate groups in LDI react with hydroxyl groups in PEGS to form a polyurethane (PU) network, providing structural integrity. Simultaneously, LDI reacts with water in the curing environment, generating amino groups and releasing carbon dioxide (CO_2_), which facilitates the development of an interconnected porous structure. Such porosity is critical for improving mass transport, nutrient exchange, and cellular infiltration, while also accelerating material degradation—thereby overcoming the inherent limitations of CPC, including its slow resorption rate and poor post‐implantation permeability. Moreover, these newly formed amino groups further react with unreacted isocyanate groups, resulting in a polyurea (PUA)‐like cross‐linking structure. Using the P2C8 formulation as a base, various isocyanate‐to‐hydroxyl ratios (denoted L or Bl_z_) were tested to assess LDI's influence on performance. To characterize the impact of LDI on pore formation, three ratios (l_1.0_, l_1.25_, and l_1.5_) were assessed. As the LDI amount increases, there showed significantly increased foaming in l_1.25_ compared to l_1.0_, while l_1.5_ shows significant volume expansion (Figure S5A, Supporting Information). These results confirm that the formation of the porous structure within the bone cement is driven by isocyanate amination. Even in the l_1.0_ formulation (1:1 isocyanate:hydroxyl), water‐induced side reactions contributed to PUA‐like network formation.

Beyond pore formation, the curing time of bone cement is a critical parameter in clinical applications. Excessively prolonged curing can elevate the risk of intraoperative bleeding, infection, and extended surgical time, thereby compromising patient outcomes. Conversely, an overly rapid setting may hinder proper handling and injection, preventing adequate filling of the bone defect and potentially leading to suboptimal fixation and increased treatment costs. Therefore, achieving an optimal balance between working time and setting time is essential to ensure both surgical operability and postoperative performance of the bone cement.^[^ ^43^ ^]^ To meet clinical demands for optimized in situ curing, the setting time of the cement was evaluated using rheometry. At physiological temperature (37 °C), uncatalyzed PEGS/CPC formulations exhibited excessively long curing times (Figure S5B, Supporting Information). To accelerate the curing process of bone cement, stannous octanoate (T‐9) was selected as a catalyst due to its ability to activate the isocyanate groups in LDI. Specifically, T‐9 interacts with the isocyanate groups, increasing their electrophilicity by polarizing the carbon atoms, thereby facilitating nucleophilic attack by the hydroxyl end groups of the polyol polymer (Figure S1C, Supporting Information).^[^ ^44^, ^45^ ^]^ Different amounts of T‐9 catalyst, 0.01%, 0.05%, and 0.1% of the total mass of bone cement, were incorporated to investigate its effect on the curing time. The introduction of T‐9 reduced the curing time of L‐PEGS/CPC to under 30 min, with a concentration‐dependent decrease observed as the catalyst content increased (Figure 2D; Figure S5C–E, Supporting Information). In contrast, B‐PEGS/CPC exhibited lower reactivity, and even with the highest T‐9 amount (0.1%), the curing time remained prolonged at ≈110 min, reflecting the limited catalytic responsiveness due to B‐PEGS's steric hindrance.

After implantation into the human body, the bone cement primarily experiences axial pressure. Mechanical testing under axial compression indicated that Ll_1.5_ exhibited the lowest compressive modulus due to excessive foaming, while both Ll_1.0_ and Ll_1.25_ struck a balance between porosity and strength (Figure 2I,J). Due to the fewer reactive sites in B‐PEGS compared to L‐PEGS, the resulting organic network density is lower, leading to inferior compressive performance of B‐PEGS/CPC under various LDI ratios compared to L‐PEGS/CPC. Similar to L‐PEGS/CPC, the compressive properties of B‐PEGS/CPC gradually decrease with increasing cross‐linker content. As expected, the incorporation of the organic network and porous structure endows the bone cement with exceptional fatigue resistance (Figure 2K,L). However, an excessive amount of LDI led to enlarged pore sizes, compromising mechanical strength. Conversely, insufficient LDI resulted in under‐cross‐linked and poorly cured networks, particularly in B‐PEGS‐based formulations. Therefore, an LDI content equivalent to 1.25 times the molar amount of hydroxyl groups was identified as optimal, achieving a favorable balance between mechanical strength and porous architecture. In addition to optimizing the concentrations of LDI and T‐9 to enhance mechanical performance, it is essential to ensure that these agents do not compromise the biocompatibility of the cement, particularly its capacity to support bone regeneration. To evaluate cytotoxicity, in accordance with the *GB/T 16 886.5‐2017* for the biological evaluation of medical devices, the L929 cell viability of bone cement formulations was assessed. According to the criteria, materials that result in cell viability below 70% of the blank control are considered potentially cytotoxic. The cell viability of L‐P2C8 containing 0.01% mass fraction of T‐9 reached close to 93%, indicating no cytotoxic response and satisfying regulatory safety requirements (Figure 2E). This observation underscores the clinical translation potential of the L‐P2C8 material, warranting further cell‐level investigations under application‐specific conditions. In contrast, the B‐P2C8 formulation with 0.1% T‐9 not only failed to cure effectively at physiological temperature but also exhibited significant cytotoxicity, highlighting the importance of precise catalyst dosing to ensure both functional and biological compatibility.

Following the optimization of the contents of PEGS, CPC, LDI, and T‐9, the formulations of L‐P2C8 and B‐P2C8 enable the bone cement to maintain injectability without leakage. When the isocyanate content in LDI is 1.25 times that of hydroxyl groups and the T‐9 content is 0.01 mass ration, both L‐PEGS/CPC and B‐PEGS/CPC exhibit a balanced combination of mechanical strength, porous structure, and biosafety. The internal structure of the bone cement after curing was further characterized using scanning electron microscopy (SEM). Both L or B‐P3C7 and L or B‐P2C8 bone cements exhibited a porous structure, attributed to the reaction between LDI and ambient moisture, leading to the generation of amino (–NH_2_) groups and the release of CO_2_.^[^ ^46^ ^]^ The low viscosity of L‐P3C7 and B‐P3C7 results in bubble aggregation, leading to the formation of large pores and a significant decrease in mechanical strength. As CPC content increased, an enhanced presence of inorganic particles was observed on the bone cement surface (Figure 2C; Figures S2–S4, Supporting Information). In contrast, the L or B‐P1C9 formulations failed to form a cohesive matrix and appeared as irregularly shaped cement particles, reflecting poor phase integration. Due to the lower hydroxyl group density in B‐PEGS, its curing time was prolonged compared to L‐PEGS, allowing CO_2_ to diffuse into the environment rather than being retained to form pores. As a result, at the same solid‐to‐liquid ratio, B‐PEGS/CPC generated fewer and smaller pores compared to L‐PEGS/CPC. This structural difference is particularly important, as extensive evidence has demonstrated that interconnected porosity enhances cell adhesion, nutrient exchange, and tissue ingrowth—key features for successful bone regeneration.^[^ ^47^, ^48^, ^49^, ^50^ ^]^

A known limitation of CPC is its tendency to disintegrate upon exposure to physiological environments, attributed to its dissolution‐recrystallization phenomenon.^[^ ^51^ ^]^ Upon contact with water, CPC powder undergoes a hydration reaction, wherein water acts as a temporary binder among inorganic particles, promoting the formation of hydrated products and reducing interparticle spacing. Once the spacing reaches a certain threshold, long‐range forces prompt polar groups on the surface of CPC to become crystallization points, initiating crystallization and achieving solidification.^[^ ^41^, ^52^ ^]^ However, this process depends on fragile calcium phosphate crystal bonding, which is vulnerable to water flow, potentially resulting in disintegration, inflammatory responses, and even thrombosis if CPC fragments enter the bloodstream post‐implantation.^[^ ^53^ ^]^ Notably, PEGS/CPC bone cement forms stable structures both before and after curing. Upon extrusion, the uncured PEGS/CPC paste can adopt irregular and anatomically complex shapes while maintaining dimensional stability in dynamic aqueous environments (Figure 2F). After curing, the PEGS/CPC remained intact under a wet environment due to the presence of organic‐inorganic interpenetrating networks (Figure 2G,H). In the presence of the cross‐linking agent LDI, both B‐PEGS and L‐PEGS form a 3D polymeric network that effectively stabilizes the CPC powder particles and inhibits the infiltration of surrounding fluids into the bone cement slurry. The PEGS matrix provides robust cohesion to the bone cement, encapsulating the inorganic phase within the organic network and thereby enhancing the cement's resistance to disintegration.

### 2.3 Hydration Reinforcement and Characterization of PEGS/CPC Composites

CPC is typically formulated from a mixture of calcium phosphate salts, primarily tetracalcium phosphate (TECP) and dicalcium phosphate anhydrous (DCPA), which undergo transformation and hardening into HA upon exposure to aqueous exposure.^[^ ^54^, ^55^ ^]^ The hydration mechanism involves a series of sequential stages: surface dissolution, crystal nucleation, water diffusion, crystal growth, and hardening. Among these, the initial three stages are kinetically rate‐limiting and critically influence the overall reaction kinetics. This hydration‐driven phase evolution directly governs the impacts the mechanical behaviors and osteogenic performance after injection, ultimately affecting the repair outcomes. Various factors have been evidenced to be involved in this procedure, particularly components and physicochemical parameters (e.g., particle size, pH of the setting solution).^[^ ^41^, ^52^ ^]^ In this study, within the organic–inorganic hybrid composite cement, the polymeric architecture and hydrophilic functional groups are hypothesized to play a critical role in modulating hydration dynamics, thereby providing a tunable strategy for optimizing cement performance.

**Figure 3:**
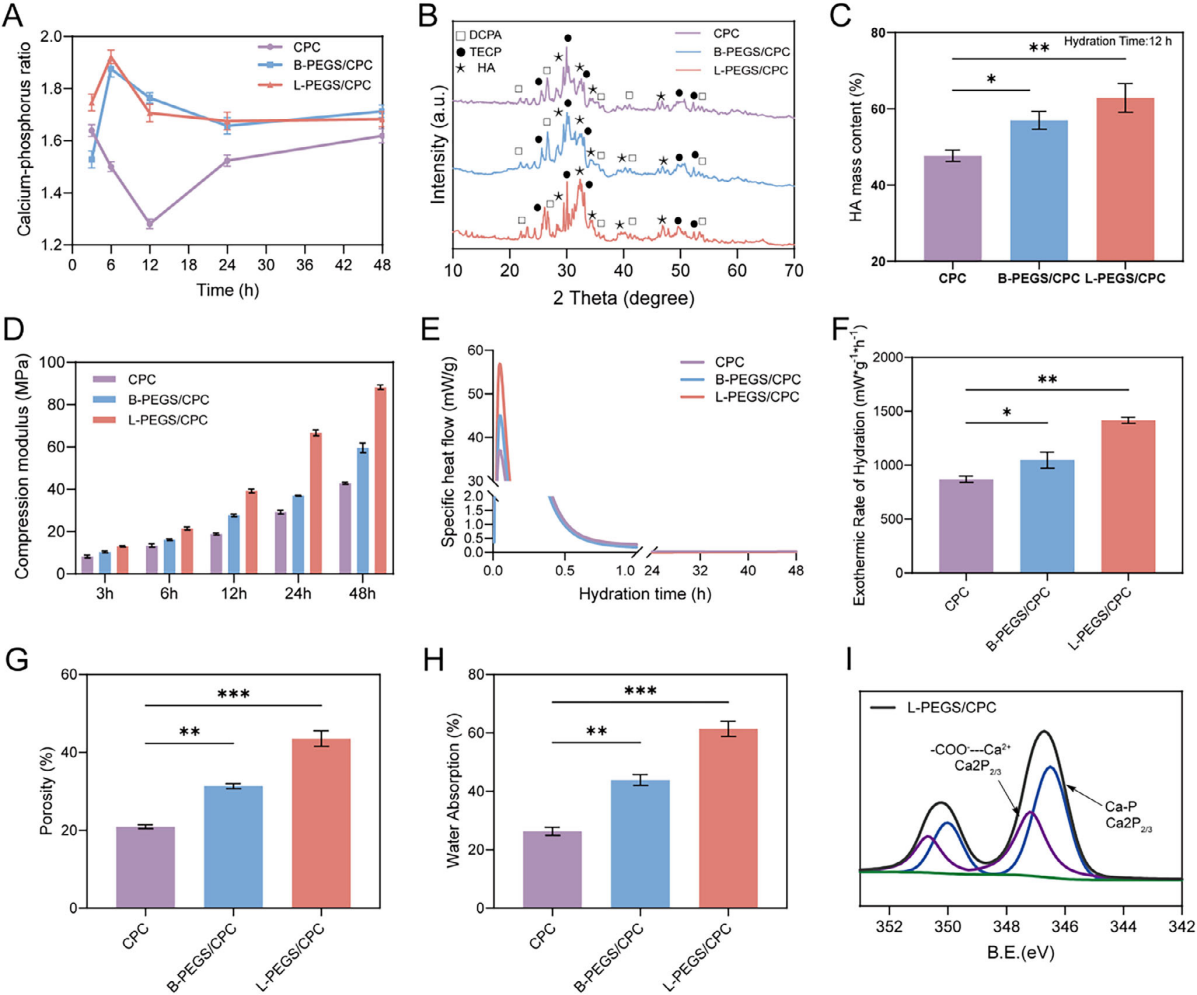
Hydration reinforcement and characterization of PEGS/CPC composites. A) Time‐dependent changes in the calcium‐to‐phosphorus (Ca/P) ratio of bone cements. The Ca/P ratio of L‐PEGS/CPC reached 1.67 at 12 h, indicating faster hydroxyapatite (HA) formation compared to B‐PEGS/CPC and CPC. B) XRD patterns of bone cement after 12 h of hydration. L‐PEGS/CPC had the highest HA peak intensity. (Cu Kα (λ = 1.5406 Å), 2θ Range: 10° to 80°, Step Size: 0.02°, Scan Speed: 1° min^−1^. The peak of DCPA, TECP, and HA are indicated with □, ●, and ★. The standard reference patterns from the International Centre for Diffraction Data (ICDD) database: HA: ICDD PDF #97‐001‐6742; TECP: ICDD PDF #97‐000‐2631; DCPA: ICDD PDF #97‐000‐2745.) C) HA mass content of bone cement after 12 h of hydration. D) Compression modulus of bone cement after different hydration time. E) The Calorimetry curves and F) exothermic rate of CPC, B‐PEGS/CPC, and L‐PEGS/CPC. G) The porosity and H) water absorption of CPC, B‐PEGS/CPC, and L‐PEGS/CPC. The superior water uptake of L‐PEGS/CPC is attributed to the synergistic effect of its high porosity and intrinsic hydrophilicity. I) XPS spectra of Ca 2p_3/2_ and 2p_1/2_ of L‐PEGS/CPC. (Data are represented as mean ± SD; *n* = 3; *p* ≥0.05 (no significant, ns), *p < 0.05; **p < 0.01; ***p < 0.001).

To evaluate the degree of hydration in CPC formulations, the calcium‐to‐phosphorus (Ca/P) ratio was measured for CPC, L‐PEGS/CPC, and B‐PEGS/CPC at various time points, using the theoretical Ca/P ratio of hydroxyapatite (Ca/P = 1.67) as a reference. As shown in **Figure** **3**A and L‐PEGS/CPC demonstrated a significantly accelerated hydration process compared to both CPC and B‐PEGS/CPC. To gain deeper insights into the hydration process, the phase composition of the bone cement was characterized at different time points using X‐ray diffractometry (XRD) analysis. During the initial stages of hydration (0–6 h), the characteristic crystalline phases of TECP (2θ = 29.8°) and DCPA (2θ = 25.9°) were predominantly observed. As hydration progressed, these peaks gradually diminished in intensity, concurrent with the emergence and growth of a broad peak at 2θ = 32°, characteristic of HA formation (Figure S6A–D, Supporting Information). After 12 h of incubation at 37 °C under 100% humidity, residual but discernible TECP and DCPA peaks were still observable, while the HA‐associated peak became more pronounced. (Figure 3B). At this time point, the transition efficiency to HA in the L‐PEGS/CPC group reached 62.85 ± 1.39%, which was significantly higher than that of CPC (47.60 ± 0.78%) and B‐PEGS/CPC (55.85 ± 1.04%) (Figure 3C). Notably, this phase transformation correlated with a progressive increase in compressive modulus, as L‐PEGS/CPC exhibited continuous enhancement in mechanical performance with extended hydration duration (from 3 to 48 h) (Figure 3D). This indicates that the rapid phase transformation during early‐stage hydration critically facilitates subsequent hydroxyapatite crystal growth and hardening, thereby significantly enhancing the mechanical robustness of the material to meet the desirable support for bone regeneration.

Herein, we posit that the distinct hydration process in polymer‐enhanced composite system primarily stem from variations in the architecture and hydrophilic motifs of polymer. In particular, the variation in hydrophilicity between L‐PEGS and B‐PEGS plays a dominant role in modulating calcium phosphate dissolution–recrystallization dynamics and regulating aqueous diffusion pathways, thereby influencing overall hydration efficiency. The heat release and exothermic rate during cement hydration critically govern the dissolution of calcium‐phosphate ions and subsequent crystal nucleation/growth on CPC surfaces. During the initial hydration phase, the multihydroxy‐rich architecture of L‐PEGS/CPC facilitates rapid cross‐linking with LDI, exhibiting significantly higher heat release and exothermic rate than B‐PEGS/CPC and pure CPC (Figure 3E,F). The rapid elevation in calcium and phosphate ion concentrations substantially enhances hydroxyapatite nucleation frequency, while higher heat promotes molecular thermal motion to accelerate crystalline phase formation. In the exothermic deceleration phase, hydration reaction decreased and HA crystallization at the material interface, inducing a characteristic transition from surface reaction control to diffusion‐controlled mechanisms.^[^ ^56^ ^]^ During the diffusion stage of water molecules in the hydration process, water molecules are absorbed and permeate into the material through its porous structure, thus initiating hydration with CPC. As shown in Figure 3G, the porosity of L‐PEGS/CPC was significantly higher compared to that of both CPC and B‐PEGS/CPC. Higher porosity in bone cement corresponds to an increased internal surface area, which enhances the contact area between CPC and water, thereby accelerating the hydration process. The incorporation of hydrophilic L‐PEGS and B‐PEGS significantly enhances the water absorption capacity of the material, thereby further reducing the hydration time of CPC (Figure 3H). In addition, X‐ray photoelectron spectroscopy (XPS) results revealed that the incorporation of PEGS resulted in a decrease in the binding energy of Ca‐P, accompanied by an increase in that of Ca‐COO^−^ (Figure 3I; Figure S6E,F, Supporting Information). In L‐PEGS/CPC, the binding energy of Ca‐COO^−^ was significantly higher than that in B‐PEGS/CPC, indicating enhanced ionic complexation of Ca‐COO^−^ in the sample and stronger interactions between the organic and inorganic networks, which collectively facilitate the hydration process. This phenomenon could be attributed to the multihydroxy architecture and COO^−^ of L‐PEGS, which enables synergistic coordination with calcium ions, thereby establishing nucleation sites that facilitate crystallization of early HA seed. Furthermore, hydrogen bonds provided by residual hydroxyl groups in the organic network, along with coordination bonds formed between Ca^2+^ and COO^−^, represent another critical factor facilitating the hydration process. Notably, the linear polymer structure of L‐PEGS imparts higher chain regularity than B‐PEGS, favoring the formation of ordered domains that guide preferential HA crystal alignment during setting.^[^ ^57^, ^58^ ^]^ This structural regularity promotes oriented crystallization, leading to faster mechanical reinforcement and improved functional performance of the cement.

Collectively, L‐PEGS/CPC orchestrates a tripartite synergy of thermodynamic, topological (connected pore structure), and chemical (coordination bonding) effects to dramatically accelerate CPC hydration, thereby overcoming the limitations of CPC systems in slow hydration and inadequate mechanical strength. L‐PEGS/CPC achieves simultaneous enhancement of hydration rate and compressive modulus, fulfilling the requirements for load‐bearing bone regeneration.

### 2.4 Exothermic, Mechanical, and Degradation Characterization of L‐PEGS/CPC Cement

Currently, PMMA bone cement is widely used in clinical practice for bone defect repair. However, its free‐radical polymerization process generates substantial heat during curing, with local temperatures reaching up to 70 °C. Notably, bone tissue experiences thermal necrosis at 50 °C,^[^ ^59^ ^]^ collagen denatures at 56 °C,^[^ ^60^ ^]^ and nerve tissue, being highly temperature‐sensitive, suffers thermal injury at 43 °C.^[^ ^61^ ^]^ While high temperatures can accelerate the setting of bone cement, they also pose a risk of irreversible cell damage and protein denaturation, potentially leading to complications that impair long‐term clinical outcomes.

**Figure 4:**
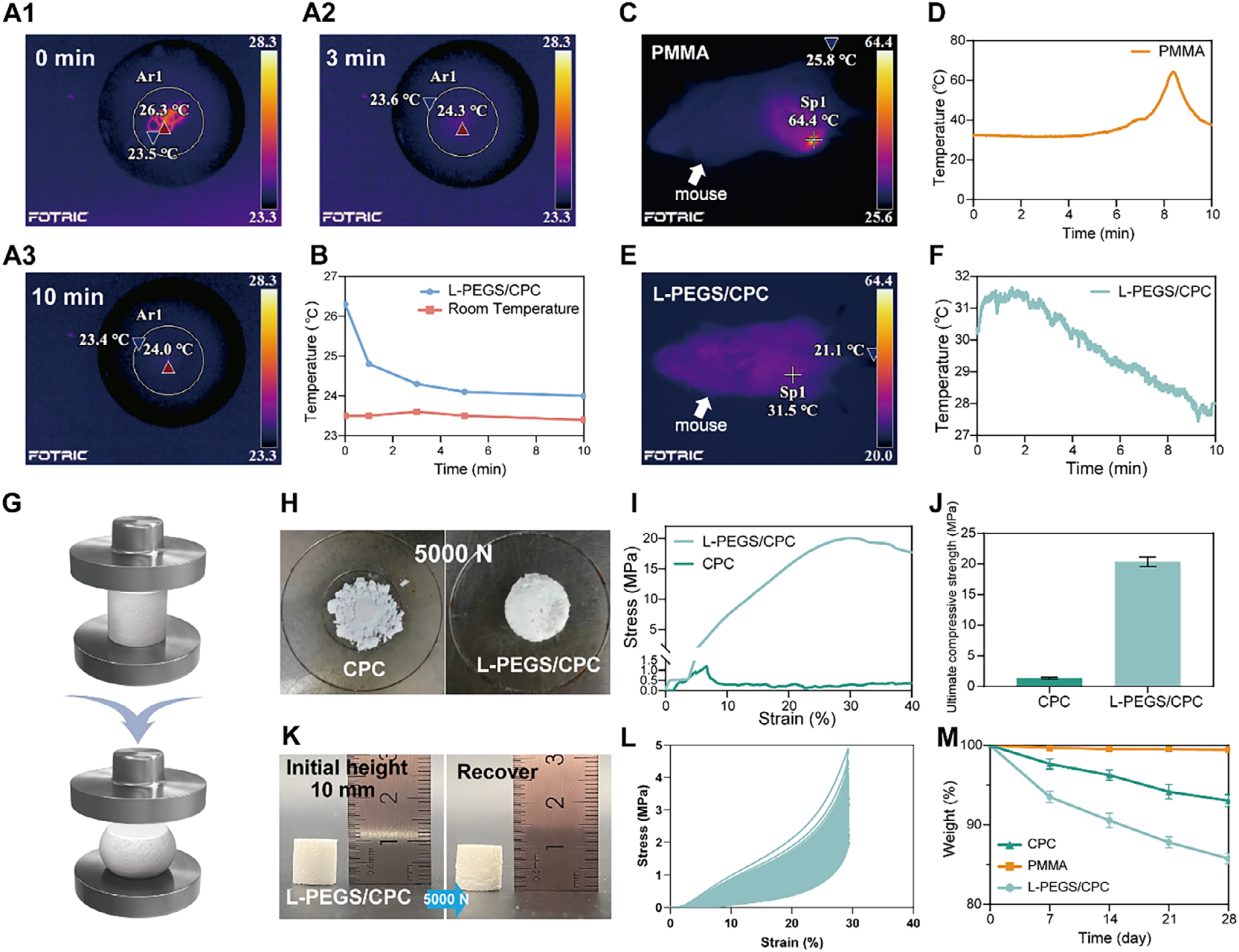
Exothermic, mechanical and degradation characterization of L‐PEGS/CPC compared to clinically used bone cement. A) Infrared thermal images of L‐PEGS/CPC after injection at 0, 3, and 10 min in vitro. L‐PEGS/CPC shows only a 2.8 °C temperature increase, attributed to shear stress and friction during injection. B) Temperature profile of L‐PEGS/CPC after injection. C) Infrared thermal images of the highest temperature of PMMA after injection in vivo. The curing temperature of PMMA can reach 64.4 °C, which may pose a risk of thermal damage to surrounding tissues. D) Temperature variation cure of PMMA after injection in vivo. E) Infrared thermal images of the highest temperature of L‐PEGS/CPC after in vivo injection. F) Temperature variation curve of L‐PEGS/CPC after in vivo injection (The temperature decline recorded in the L‐PEGS/CPC group can be ascribed to the concurrent impact of the low ambient temperature and drug‐induced anesthesia.). G) Schematic representation of L‐PEGS/CPC compression experiment. H) Visual comparison of CPC and L‐PEGS/CPC specimens after compression testing. I) Compressive stress–strain curves and J) ultimate compressive strength of CPC and L‐PEGS/CPC. K) Digital photos of elastic recovery in L‐PEGS/CPC following compression. L) Cyclic compressive loading–unloading curves of L‐PEGS/CPC over 1000 cycles. M) Degradation profiles of PMMA, CPC, and L‐PEGS/CPC over 28 days in vivo.

To assess the exothermic behavior of L‐PEGS/CPC during curing, thermal imaging was performed using an infrared camera. Owing to the shear forces exerted during the injection process, the initial temperature of the bone cement was elevated by ≈2.8 °C above ambient, reaching a peak of 26.3 °C immediately post‐injection. while some heat generation is unavoidable, the observed temperature rise remained within a biologically safe range and posed no risk to surrounding tissues. Subsequently, the temperature gradually declined, reaching only 0.6 °C above ambient within 5 min. At 10 min post‐injection, the temperature stabilized at 24.0 °C, indicating that equilibrium with the ambient temperature had not been reached (**Figure** **4**A,B; Figure S7A, Supporting Information). This residual heat might originate from the curing reaction, not only indicate the persistent reaction but also actively accelerate the initial hydration of CPC. To further compare the exothermic characteristics of L‐PEGS/CPC and PMMA in vivo, real‐time thermographic monitoring was performed post‐implantation in murine femoral defect models. In contrast to the mild exothermic response observed with L‐PEGS/CPC, PMMA bone cement exhibited a pronounced heat release, with the implantation site temperature rising to 64.4 °C (Figure 4C,D). L‐PEGS/CPC exhibited no profound exothermic phenomenon during curing in vivo, thereby reducing thermal damage to surrounding bone tissues (Figure 4E,F).

To investigate the mechanical effects arising from the composite of the organic network of L‐PEGS and the inorganic network of CPC, compression tests were conducted on L‐PEGS/CPC and CPC under a pressure of 5000 N (Figure 4G). After hydration, CPC exhibited brittleness, fracturing into fragments under pressure, whereas hydrated L‐PEGS/CPC was gradually compacted without fragmenting (Figure 4H). Notably, L‐PEGS/CPC exhibits a remarkable enhancement in mechanical performance, achieving a 16.7‐fold increase in ultimate compressive strength (20.37 ± 0.59 MPa) compared to unmodified CPC (1.22 ± 0.12 MPa) (Figure 4I,J). This was attributed to the presence of L‐PEGS, which provides sufficient cohesion to CPC, simultaneously elevating compressive strength while effectively preventing its brittleness‐induced fragmentation. Although L‐PEGS/CPC gradually degrades in physiological environments, it retains excellent mechanical performance. This is attributed to the sequential degradation mechanism, wherein the organic network progressively degrades while the hydrated inorganic CPC or HA matrix continues to provide structural support. In contrast to the nearly non‐degradable nature of PMMA, L‐PEGS/CPC not merely maintains sufficient mechanical integrity during the early stages of implantation, but allows biodegradation, thereby offering space for new bone ingrowth. Currently, clinically used bone cements such as PMMA and CPC are rigid materials, and development on elastic bone cements remains limited. To further evaluate the elasticity of L‐PEGS/CPC bone cement, a compressive fatigue test was conducted. A cylindrical L‐PEGS/CPC specimen with an initial height of 10 mm was compressed to a height of 3 mm under a load of 5000 N. Upon removal of the pressure, the sample recovered its original shape within 30 s (Figure 4K). After 1000 cycles of compressive loading at 30% strain, L‐PEGS/CPC retained its original morphology, demonstrating exceptional fatigue resistance (Figure 4L). Such elastic behavior allows L‐PEGS/CPC to store mechanical energy under excessive stress, thereby preventing stress‐induced secondary fractures in surrounding bone tissue—a common issue associated with high‐modulus materials. In addition to mitigating stress‐related damage, the elastic recovery of L‐PEGS/CPC also reduces the risk of brittle fracture and fragmentation observed in CPC, minimizing the formation of debris that could otherwise induce local inflammation and compromise long‐term implant stability.^[^ ^62^ ^]^ In a dynamic bone growth environment, the toughness and compliant mechanical properties of L‐PEGS/CPC not only provide sufficient mechanical support during the early postoperative period but also minimize the risk of secondary complications.

In addition to excessive exothermicity and mechanical brittleness, the lack of biodegradability remains a major limitation of current clinically available bone cements. Their inability to undergo natural metabolic clearance and facilitate new bone ingrowth significantly hampers long‐term regenerative outcomes, particularly in younger patients or in cases requiring active bone remodeling.^[^ ^17^ ^]^ As shown in Figure 4M, quantitative degradation analysis indicated distinct mass retention profiles after 28‐day in vivo implantation. PMMA remained virtually intact after 28 days, retaining 99.46 ± 0.13% of its original mass. CPC exhibited minimal degradation, with 93.08 ± 0.58% mass retention, indicating limited biodegradability. In contrast, L‐PEGS/CPC demonstrated a higher and more controlled degradation profile, retaining 84.71 ± 0.44% of its initial mass. The non‐degradable nature of PMMA hinders its replacement by newly formed bone, thereby obstructing the natural repair process. Meanwhile, PMMA lacks intrinsic bioactivity and cannot chemically bond with host bone tissue or support osteogenic cell attachment and proliferation, relying solely on mechanical interlocking for fixation. CPC, while biodegradable, features a densely packed structure that limits fluid infiltration and ionic exchange, thereby slowing the surface dissolution–reprecipitation process. In addition, its poorly interconnected porosity hampers the diffusion of degradation byproducts (e.g., Ca^2^⁺ and PO_4_^3−^), further inhibiting bulk degradation. In this study, L‐PEGS/CPC exhibits demonstrates a favorable biodegradation profile, which is controllable to not merely preserve mechanical integrity during the early healing phase, but allows space for new bone ingrowth, supporting effective long‐term regeneration.

In summary, L‐PEGS/CPC overcomes the high exothermic issue associated with PMMA curing, exhibits superior fatigue resistance not observed in PMMA or CPC, and undergoes gradual degradation accompanied by new bone growth, demonstrating significant clinical advantages and promising potential for practical applications.

### 2.5 In Vitro Cell Behaviors on L‐PEGS/CPC Bone Cement

**Figure 5:**
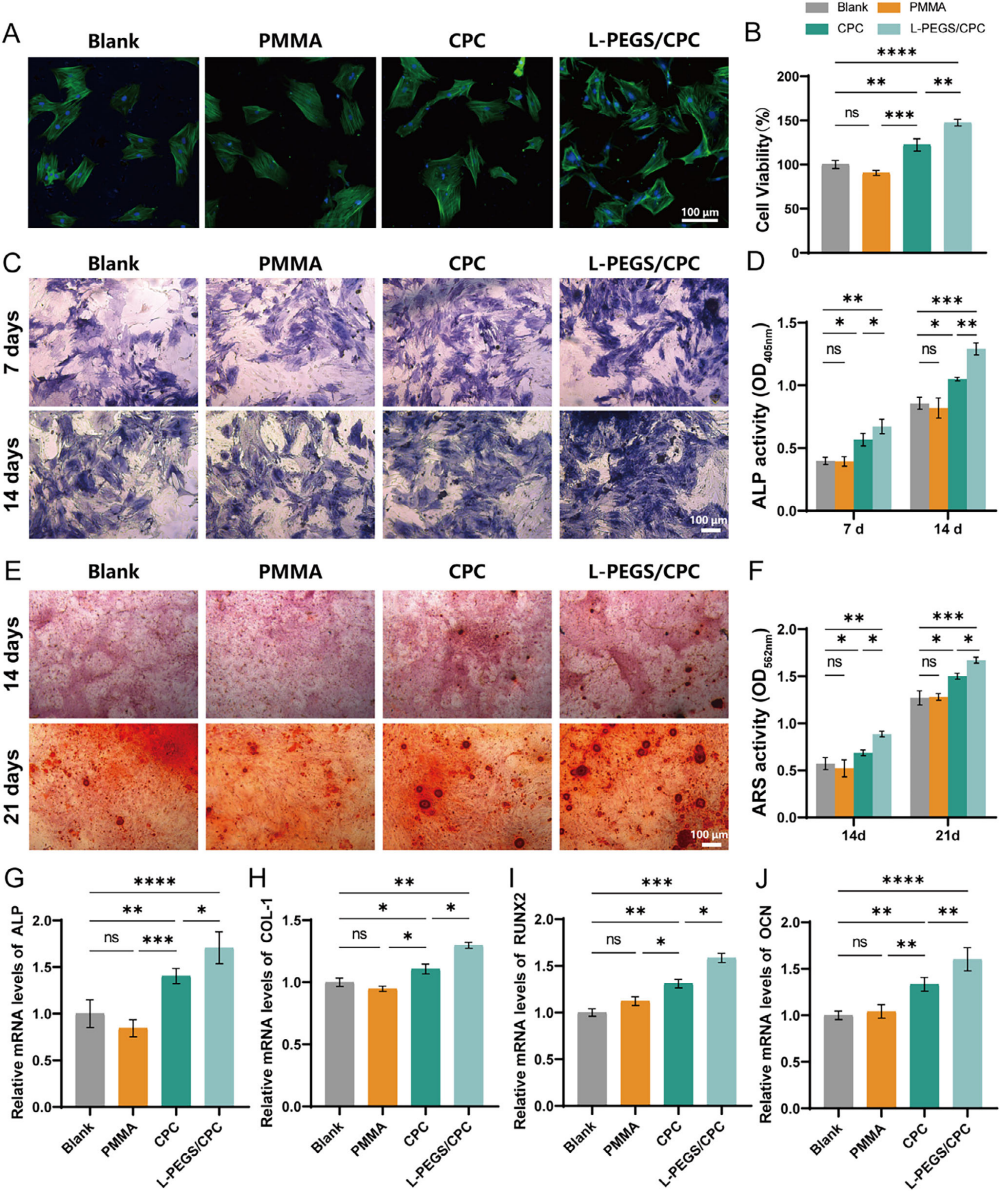
In vitro cell behaviors on L‐PEGS/CPC bone cement. A) Fluorescent micrographs of rBMSCs adhered on different bone cements after staining with FITC, DAPI and Sudan Black B. (To promise cell immobilization on the material scaffold, glutaraldehyde was selected as the fixation agent, with Sudan Black B employed to quench autofluorescence originating from both the glutaraldehyde and the material itself.) (Scale bar:100 µm). B) The cell viability of CPC, L‐PEGS/CPC, and PMMA toward BMSCs. (The L‐PEGS/CPC group exhibited proliferation‐promoting characteristics when against other group.) C) ALP staining of BMSCs co‐cultured with CPC, L‐PEGS/CPC, and PMMA for 7 days and 14 days. (Scale bar:100 µm). D) Quantification of ALP activity on day 7 and day 14. E) ARS staining of BMSCs co‐cultured with CPC, L‐PEGS/CPC, and PMMA for 14 days and 21 days. (Scale bar:100 µm). F) Quantification of ARS activity on day 14 and day 21. qRT‐PCR analysis of G) ALP, H) COL‐1, I) RUNX2, and J) OCN in BMSCs with the treatment of CPC, L‐PEGS/CPC and PMMA for 7 days. (Data are represented as mean ± SD; *n* = 5; *p* ≥0.05 (no significant, ns), *p < 0.05; **p < 0.01; ***p < 0.001, ****p < 0.0001).

Initial cell adhesion to the cement surface plays a critical role in regulating subsequent cellular behaviors, including spreading, proliferation, and differentiation —particularly the lineage specification of recruited stem cells. Previous studies have demonstrated that a well‐spread, flattened cell morphology is favorable for promoting osteogenic differentiation of stem cells, which is essential for desirable bone healing.^[^ ^63^, ^64^ ^]^ Notably, stiffer surface preferentially drives osteogenic differentiation, whereas soft surface foster cellular adhesion and proliferative expansion.^[^ ^65^, ^66^ ^]^ To assess the early‐stage cellular response to the implanted bone cements, BMSCs were seeded onto L‐PEGS/CPC surfaces and incubated for 24 hours, with commercially available PMMA and CPC bone cement used as benchmarks. The cell adhesion capability of the cements assessed through cytoskeletal fluorescence staining and quantitatively evaluated using the MTT assay. As shown in **Figures** **5**A and S8G (Supporting Information), L‐PEGS/CPC composite demonstrates enhanced cellular adhesion and proliferation compared to CPC and PMMA, attributable to the soft surface imparted by the L‐PEGS modification. Quantitative analysis of cell viability toward BMSCs (Figure 5B; Figure S8H, Supporting Information) indicated significant discrepancy between the L‐PEGS/CPC and both PMMA and CPC group, indicating that L‐PEGS/CPC is non‐cytotoxic and supports normal cell attachment during the initial stages.

ALP activity is widely recognized as an early‐stage marker of osteogenic differentiation in stem cells, owing to its role in hydrolyzing organic phosphoesters and facilitating calcium phosphate deposition during bone formation.^[^ ^67^, ^68^ ^]^ In this study, both ALP staining and activity assays were initially performed to evaluate the osteogenic differentiation of BMSCs cultured on L‐PEGS/CPC, CPC and PMMA cements. As shown in Figure 5C and Figure S9A (Supporting Information), the L‐PEGS/CPC and CPC exhibited significantly enhanced ALP staining intensity at both 7 and 14 days compared to the PMMA and blank control groups.^[^ ^69^, ^70^ ^]^ In contrast, PMMA bone cement showed no significant difference in ALP activity compared to the blank group, consistent with its known biological inertness and lack of osteoinductive properties. Quantitative analysis of ALP activity (Figure 5D) further confirmed these observations. At day 7 and day 14, the L‐PEGS/CPC group exhibited 1.70‐ and 1.45‐fold higher ALP activity, the CPC group exhibited 1.41‐ and 1.22‐fold higher ALP activity, respectively, compared to the blank control, while PMMA showed no significant enhancement. Notably, the ALP activity of L‐PEGS/CPC was significantly higher than that of CPC alone at both time points, highlighting the positive influence of L‐PEGS incorporation in promoting early‐stage osteogenic differentiation of BMSCs. Similar to ALP results, Alizarin Red S (ARS) staining indicated the most substantial mineral deposition in the L‐PEGS/CPC group at both 14 and 21 days (Figure 5E; Figure S9B, Supporting Information). As shown in Figure 5F, quantitative analysis of ARS staining revealed that L‐PEGS/CPC composites exhibited 1.55‐ and 1.29‐fold enhancements in mineralization activity at day 7 and day 14, respectively, compared to blank controls, whereas CPC alone demonstrated more modest increases of 1.20‐ and 1.18‐fold. In contrast, PMMA showed negligible osteogenic potential throughout the observation period. To further substantiate these phenotypic observations at the molecular level, real‐time RT‐qPCR was conducted to quantify the expression of key osteogenic genes, including ALP, type I collagen (COL I, main content of bone extracellular matrix), Runt‐related transcription factor 2 (RUNX 2, a key transcript factor for bone formation), and osteocalcin (OCN, a late marker for osteogenic differentiation) (Figure 5G–J; Figure S8A–D, Supporting Information). The L‐PEGS/CPC group exhibited the highest upregulation of all four genes compared to the blank and PMMA groups, further confirming its superior osteoinductive capability.

The limited bioactivity of conventional PMMA cement inherently restricts its therapeutic potential, primarily due to the absence of bioactive components necessary for initiating bone regeneration. Similarly, the insufficient osteoactivity of CPC, combined with the inherent bioinertness of polymeric composites—which, despite enhancing mechanical properties, fail to improve bioactivity—significantly limits the clinical applicability and therapeutic efficacy of CPC/polymer composites. Interestingly, the incorporation of L‐PEGS into CPC markedly enhanced osteogenic differentiation of BMSCs. While the underlying mechanisms remain to be fully elucidated, we proposed that that the observed effects are attributable to both surface physicochemical characteristics and chemical composition of the L‐PEGS/CPC combination. Specifically, the enhanced hydrophilicity and modulated surface architecture resulting from soft–hard phase interactions could synergistically promote cell adhesion proliferation, and osteogenic commitment (Figure S8E,F, Supporting Information). Moreover, the incorporation of L‐PEGS accelerates cement degradation, thereby enhancing the release of calcium and phosphate ions, which in turn contributes to the observed improvement in osteogenic responses. Given the complex interplay between surface topography, mechanical compliance, and chemical composition, the specific contribution of each parameter to the overall cellular behavior is worth further systematic investigation.^[^ ^71^ ^]^

### 2.6 In Vivo Bone Regeneration

**Figure 6:**
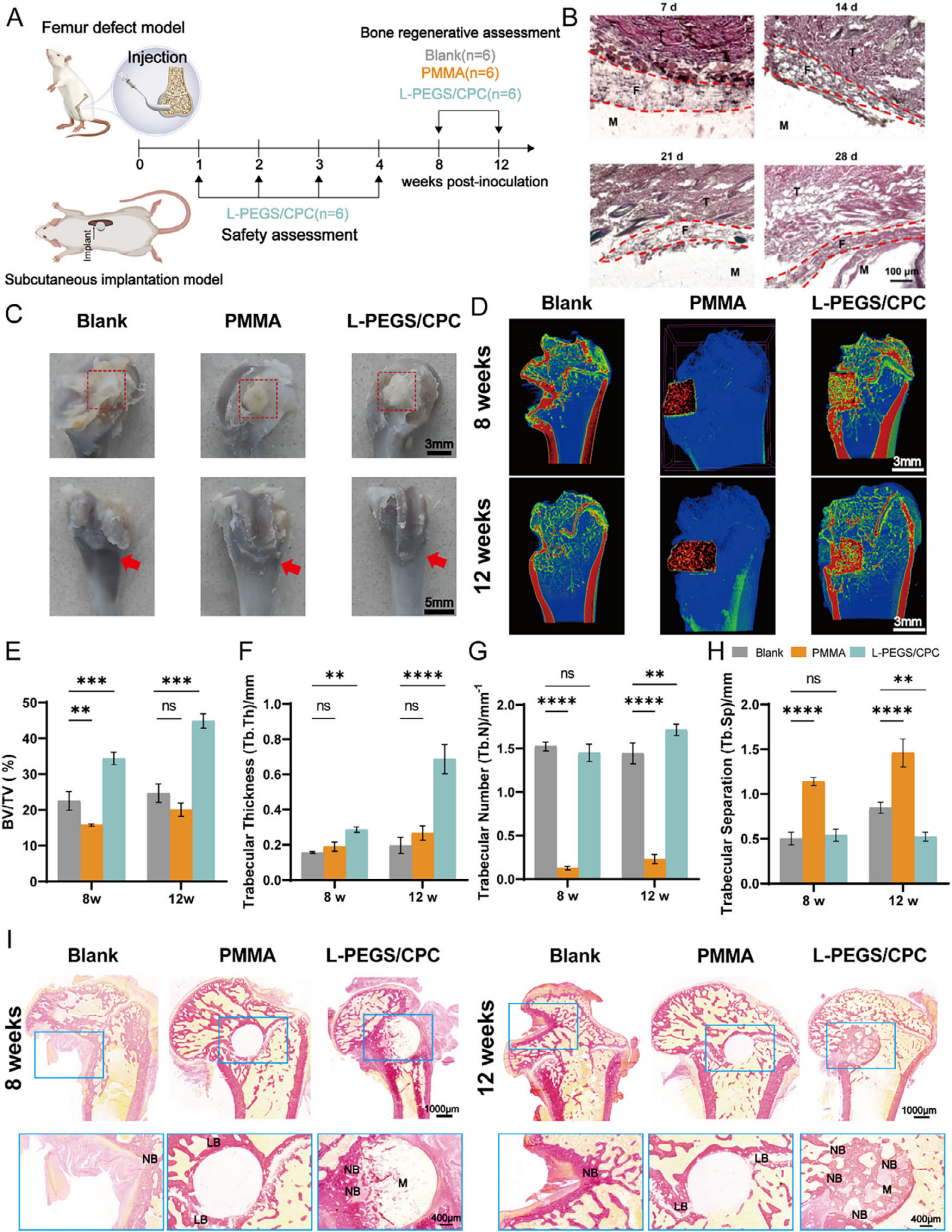
In vivo bone regeneration. A) Schematic representation of rodent model experiment. B) Histologic sections of L‐PEGS/CPC bone cement for safety assessment. (T: tissue; F: inflammatory fiber; M: material). (Scale bar: 100 µm). C) Photograph of femoral defect repair in rats at 12 weeks of material implantation. (Scale bar: 3 and 5 mm). D) 2D micro‐CT images of the femur treated with the PMMA and L‐PEGS/CPC for 8 and 12 weeks (In the PMMA group, the visualization of the bone structure was compromised to better resolve the residual material.). (Scale bar: 3 mm). Quantification of E) bone volume fraction (BV/TV; %), F) trabecular thickness (Tb.Th, mm), G) trabecular number (Tb.N) and H) trabecular separation/spacing (Tb.Sp, mm) derived from micro‐CT at 8 weeks and 12 weeks. I) Representative Van Gieson (VG) staining images of the defect in the femur. M: residual materials; LB: lamellar bone; NB: new bone. (The extraction of PMMA caused dimensional contraction of the osseous defect region during subsequent dehydration, clearing, and embedding procedures.). (Scale bar: 1000 and 400 µm). (Data are represented as mean ± SD; *n* = 6; *p* ≥0.05 (no significant, ns), *p < 0.05; **p < 0.01; ***p < 0.001, ****p < 0.0001).

In prior in vitro studies, L‐PEGS/CPC demonstrated significantly enhanced osteogenic differentiation compared to commercial CPC and PMMA, as indicated by elevated alkaline ALP and Alizarin Red S ARS activity, along with the upregulation of key osteogenic markers. These results highlight the huge bioactivity and osteoinductive potential of the L‐PEGS/CPC composite. We further evaluated the long‐term bone regeneration capacity, in vivo biocompatibility and degradation of L‐PEGS/CPC by subcutaneous implantation and femoral defect model in rats (**Figure** **6**A). The host responses and degradation behavior in vivo were initially investigated through subcutaneous dorsal implantation of the samples on rats. As shown in Figure 6B, after implantation of L‐PEGS/CPC composites in rats, the fibrous capsule thickness demonstrated a significant reduction from 156 ± 2.4 µm at 1 week to 71 ± 1.3 µm at 4 weeks. In accordance with *ISO 10993‐6:2016* evaluation criteria, L‐PEGS/CPC exhibited outstanding biocompatibility with progressive host tissue adaptation. Throughout the observation period, all animals exhibited normal activity and feeding behavior without any signs of systemic toxicity or adverse reactions, indicating the excellent long‐term in vivo biocompatibility of the material.

Following the confirmation of long‐term in vivo safety profiles, a rat femoral defect model was established to further evaluate the bone regeneration capacity different cement groups, specifically the Sham, PMMA, and L‐PEGS/CPC groups. Two time points—8 and 12 weeks were selected for comparative evaluation of L‐PEGS/CPC against commercial PMMA. As shown in Figure S10A (Supporting Information), the L‐PEGS/CPC exhibited desirable maneuverability comparable to clinically‐used PMMA and achieved complete defect filling. At both 8 and 12 weeks post‐implantation, the Sham group displayed extensive necrosis near the femoral condyle, likely due to inadequate mechanical support and persistent inflammation. Both L‐PEGS/CPC and PMMA groups maintained femoral structural integrity, attributed to their sufficient mechanical strength. Notably, L‐PEGS/CPC released minimal heat during implantation and demonstrated excellent integration with the surrounding bone tissue at 8 weeks, indicating that its low exothermic profile does not compromise tissue repair (Figure 6C). In contrast, the significant heat released by PMMA during implantation resulted in thermal damage to adjacent tissues and poor osseointegration, as evidenced by a persistent gap between the PMMA implant and host bone.

Micro‐CT analysis further confirmed bone regeneration in three groups. In the Sham group, large unhealed gaps were evident, while PMMA showed limited bone integration due to its bioinert nature. In contrast, L‐PEGS/CPC demonstrated intimate integration with new bone tissue (Figure 6D). Quantitative parameters, including bone volume/tissue volume (BV/TV), trabecular number (Tb.N), trabecular thickness (Tb.Th) and Trabecular Separation/Spacing (Tb.Sp), further confirmed these findings (Figure 6E–H; Figure S10B–D, Supporting Information). More specifically, at 8 weeks, the BV/TV for L‐PEGS/CPC reached 34.39 ± 0.83%, significantly higher than that of the Sham (22.54 ± 1.03%) and PMMA (15.80 ± 0.21%) groups. By 12 weeks, BV/TV in the L‐PEGS/CPC group increased by 1.3‐fold, whereas minimal changes were observed in the Sham groups. Tb.Th (Figure 6F) in the L‐PEGS/CPC group increased from 0.29 ± 0.43% mm at 8 weeks to 0.68±1.26% mm at 12 weeks, which are significantly higher than the Tb.Th in Sham and PMMA groups. Similarly, at 12 weeks, the Tb.N (Figure 6G) of PEGS/CPC group was significantly higher than the Blank and PMMA group. All these results indicate that L‐PEGS/CPC can effectively promote the thickening of trabecular bone and the densification of new bone compared to the Sham and PMMA groups. Notably, Tb.Sp (Figure 6H) was minimal in L‐PEGS/CPC, indicating denser and more mature trabecular bone architecture compared other groups.

Histological evaluation using Van Gieson (VG) staining further substantiated the regenerative potential of the implanted cements (Figure 6I). In the Sham group, substantial bone deformity and limited repair were observed, showing no substantial osseous regeneration by week 12. Although PMMA provided mechanical support, its inertness hindered integration, with new bone and extensive fibrous tissue forming only around the periphery of the material. In contrast, the L‐PEGS/CPC group, conversely, demonstrated structurally ordered and intense red collagen staining in the bone defect area, suggestive of advanced osteogenesis and bone matrix maturation. L‐PEGS/CPC promoted extensive new bone in growth through its porous matrix, with a continuous interface devoid of fibrous tissue gaps. Finally, histopathological examination of major organs revealed no discernible pathological alterations in the heart, liver, spleen, lungs, or kidneys (Figure S11A,B, Supporting Information), demonstrating the absence of systemic toxicity and excellent biocompatibility of L‐PEGS/CPC.

These results suggest that the interconnected porous architecture of L‐PGS/CPC facilitates robust infiltration of nascent tissue and vasculature, while its synergistic surface hydrophilicity actively recruits stem cells and osteoprogenitors, enhancing cellular adhesion and proliferation to create an osteoinductive niche. As L‐PEGS/CPC undergoes accelerated in situ hydration at the defect site, the resulting HA deposition further augments osteogenic differentiation and mineralization. Concurrently, the material's degradability generates void spaces for rapid osseous ingrowth, with the degradation process concomitantly liberating calcium and phosphate ions from CPC to amplify bone regeneration through a self‐reinforcing mechanism. L‐PEGS/CPC bone cement offers clinical advantages in bone defect repair owing to its high elasticity, degradability, absence of heat generation, and osteoinductive properties.

### 2.7 Clinical Perspectives

Minimally invasive therapies are widely recognized as the preferred approach for bone augmentation due to their reduced surgical trauma and accelerated postoperative recovery. However, significant challenges remain with currently available injectable bone substitutes, primarily owing to the inability to simultaneously meet key clinical requirements—namely, manipulation feasibility, mechanical supports, desirable bioactivity and safety. For now, clinical application highly relies on two main categories of bone cements: PMMA and CPC. As shown in Scheme 1C, PMMA provides excellent mechanical support and rapid setting; however, its pronounced exothermic reaction and inherent bioinertia induce peri‐implant tissue necrosis and impede osseointegration. In contrast, CPC is favored for its biomimetic formulation and superior biocompatibility, yet its intrinsic brittleness fundamentally limits load‐bearing capacity in dynamic physiological environments. Disintegration in CPC compromises the implant‐bone interfacial integrity, impairing osseointegration efficiency, while the resulting micro‐scale debris may trigger chronic inflammatory response. To overcome these clinical limitations, we developed a novel injectable, hydration‐enhanced composite cement composed of L‐PEGS and CPC. that synergistically overcomes CPC's fragility while improving the mechanical properties and biological activity of materials. Distinct from conventional polymer‐modified CPC systems, L‐PEGS demonstrates exceptional phase homogeneity while addressing inherent drawbacks of CPC through its simple formulation. The unique linear architecture and pronounced hydrophilicity of L‐PEGS synergistically accelerate cement hydration kinetics, enabling rapid development of mechanical strength that meets critical load‐bearing requirements for osseous defects, thereby facilitating enhanced bone regeneration. This innovative formulation maintains optimal injectability while achieving rapid physiological‐temperature curing—a critical advancement for clinical translation.

Comprehensive characterization reveals that the L‐PEGS/CPC cement achieves an optimal balance between mechanical stability, interconnected porosity, and structural support, while demonstrating remarkable osteoinductive potential in cellular assays. Notably, in a critical‐sized femoral defect model, the L‐PEGS/CPC group exhibited superior bone regeneration outcomes, highlighting its clinical translation potential. This injectable, hydration‐enhanced L‐PEGS/CPC bone cement represents a promising clinical solution, combining facile operability with biomechanical compatibility and enhanced bioactivity, which address critical needs in bone defect reconstruction.

## 3 Conclusion

In this study, we developed an injectable, hydration‐reinforced, and porous‐forming composite bone cement composed of linear polyether glycerol sebacate (L‐PEGS) and calcium phosphate cement (CPC). It was demonstrated that L‐PEGS/CPC exhibits excellent injectability and operability, which could achieve solidification within 30 min in vivo. The incorporation of the L‐PEGS organic network significantly reinforced the intrinsic mechanical limitations of conventional CPC, preventing fragmentation under compressive stress and imparting the composite with elasticity, fatigue resistance, and anti‐collapsibility. Most importantly, the linear polyhydroxy backbone of L‐PEGS initiated a self‐reinforcing cross‐linking reaction that was synchronized with CPC hydration into hydroxyapatite. This hydration‐induced reinforcement was driven by the high density of hydroxyl groups and the hydrophilic nature of L‐PEGS, which collectively accelerated the hydration kinetics. Concurrently, the cross‐linking reaction generated in situ carbon dioxide bubbles, forming a porous microarchitecture that not merely enhanced cement degradability but also facilitated ion exchange, nutrient diffusion, and new bone ingrowth. In vitro and in vivo evaluations further confirmed that L‐PEGS/CPC significantly improved osteogenic outcomes compared to CPC and PMMA. Collectively, these findings demonstrate that this injectable, hydration‐driven, and self‐reinforcing bone cement addresses critical limitations of current bone graft materials and holds promising translational potential for clinical bone repair applications.

## 4 Experimental Section

### Materials

All chemical reagents could be used directly without further purification. N, N‐dime‐thylformamide (DMF), LDI, commercial‐grade polyurethane (PU) and other chemical supplies were purchased from Shanghai Aladdin Bio‐Chem Technology Co., Ltd. CPC powder was purchased from Shanghai Rebone Biomaterials Co., Ltd. The high‐density polyethylene (HDPE) was bought from Shanghai Maclin Biochemical Technology Co., Ltd. Other common organic reagents such as sebacic acid were purchased from Shanghai Titan Technology Co., Ltd.

### Synthesis of Branched‐PEGS Polymer

The synthesis of branched‐PEGS (B‐PEGS) prepolymer was prepared via a two‐step polycondensation reaction according to the previous studies. Briefly, PEG (20 g, 0.4 equiv) was first vacuum heated (90 °C) to be molten and then mixed with 0.05 mol sebacic acid (10.11 g, 1 equiv) at 130 °C under Argon flow for 2 h. Subsequently, the reaction was maintained at 150 °C under a vacuum of 60 mTorr for a duration of 4 h. In the second step, glycerol (7.36 g, 1.6 equiv) and sebacic acid (18.20 g, 1.8 equiv) were introduced into the reaction flask under an argon atmosphere. The mixture was then subjected to further reaction at 130 °C under a vacuum of 60 mTorr for a period of 48 h. The final product was obtained following dialysis using a 3500 Da molecular weight cutoff membrane, and subsequently lyophilized for further utilization.

### Synthesis of L‐PEGS Polymer

L‐PEGS were synthesized via Bis(tetra‐butylammonium) sebacate (TBAS)‐induced epoxy ring‐opening reaction. Sebacic acid (10.11 g, 1 equiv.), PEGDGE (25 g, 1 equiv.), and the catalyst TBAS (0.21 g, 0.006 equiv.) were mixed and homogeneously dissolved in 100 mL of anhydrous DMF in a glovebox filled with high‐purity nitrogen. The flask was connected to a Schlenk line and heated to 100 °C in an oil bath. After 72 h of continuous stirring under a nitrogen atmosphere, the mixture was precipitated in pre‐cooled ethyl ether and dried overnight at ambient temperature under vacuum to obtain crude PEGS. The crude PEGS was dialyzed (MWCO: 3500; Viskase, USA) against water for 24 h and ethanol for 24 h to remove residual small molecules. The resultant purified PEGS solution was dried under vacuum.

### Characterization of B‐PEGS and L‐PEGS

The B‐PEGS and L‐PEGS were analyzed by nuclear magnetic resonance (NMR) spectroscopy (Bruker AVANCE III 600, Switzerland) in CDCl3 and attenuated total reflectance–Fourier transform infrared spectroscopy (ATR–FTIR, Nicolet 6700, USA). The molecular weight and polydispersity index of B‐PEGS and L‐PEGS were determined by gel permeation chromatography (GPC) using a Shimadzu Prominence HPLC instrument (Kyoto, Japan). The melting and crystallization behaviors of B‐PEGS and L‐PEGS were carried out on a differential scanning calorimeter (TA Q‐1000, USA).

### Fabrication of Injectable CPC Bone Cement

The injectable CPC cement is primarily composed of calcium phosphate cement (CPC) powder and ultrapure water, with the CPC powder itself consisting of mixture of tetracalcium phosphate (TECP) and dicalcium phosphate anhydrous (DCPA). For its preparation, precisely weighed quantities of the CPC powder and ultrapure water are combined and physically mixed to form the injectable cement paste.

### Fabrication of Injectable PEGS/CPC Bone Cement

The injectable PEGS/CPC bone cement mainly consists of PEGS, CPC, stannous octanoate (T‐9) and LDI. Specifically, L‐PEGS and B‐PEGS were respectively weighed and thoroughly mixed with CPC under vigorous stirring to achieve a homogeneous blend. Subsequently, the cross‐linker LDI and the catalyst stannous octanoate are separately added to the mixture. Finally, the bone cement paste was physically mixed thoroughly. By screening different proportions of PEGS, CPC, LDI, and stannous octanoate, the formulation for injectable PEGS/CPC bone cement was ultimately determined.

### Characterization of Injectable PEGS/CPC Bone Cement

After the curing of bone cement, its organic components form insoluble and non‐melting cross‐linking structures, and their chemical structures in the organic phase were determined by solid state nuclear magnetic resonance (^13^C NMR) spectroscopy (Bruker Ascend 500 MHz, Bruker Corporation, Switzerland). The obtained data was processed and analyzed by MestReNova NMR software. The Fourier transform infrared spectroscopy (FTIR, Nicolet 5700, Thermo Scientific, USA) of the bone cement material was recorded in the range of 500–4000 cm^−1^. Surface morphology of bone cement with different composite ratios was observed by scanning electron microscopy (SEM, Hitachi, JPN). Thermogravimetric analysis (TGA, NETZSCH STA409PC) was conducted under a nitrogen atmosphere with a heating rate of 10 °C per minute to determine the composite ratio of organic and inorganic phases in the bone cement. Energy Dispersive Spectrometer (EDS, Falion 60S, EDAX Corporation, USA) was employed to analyze the distribution of surface elements in the samples. Thermal images of samples at different time intervals were captured using Infrared Thermal Camera (FOTRIC 220s), and the images were analyzed by FOTRIC AnalyzIR software. Water was added to a small bowl made of polytetrafluoroethylene (PTFE), into which the bone cement paste was then poured to examine the anti‐washout properties of the sample before curing. The bone cement paste was injected into a specific shape on PTFE sheet and then cured in constant temperature and humidity chamber at 37 °C. The resistance to disintegration of the cured samples was evaluated using water flow impact.

### Mechanical Assessment of Injectable PEGS/CPC Bone Cement

To systematically evaluate the mechanical properties of bone cement with varying cross‐linker content, compressive strength and fatigue resistance of the bone cement were tested using an electronic universal testing machine (SANS CMT2503). The pre‐prepared bone cement paste was thoroughly mixed and subsequently injected into cylindrical molds. The molds were made of polytetrafluoroethylene (PTFE) material, with each cylinder having a diameter of 6 mm and a height of 10 mm. The appropriate cylindrical samples (with no surface cracks or defects) were placed at the center of the compression platen, and the sample parameters were inputted. A 5000 N sensor was selected, and the compression rate was set to 10 mm min^−1^ for testing. The compressive strength of bone cement was evaluated through stress‐strain curves. The fatigue resistance of bone cement was assessed through cyclic compression experiments with a compression rate set at 10 mm min^−1^. Raising the compressive stress to 12 MPa and then releasing it to 0 MPa was considered one complete cycle. Each sample undergoes 10 cycles of compression, with stress‐strain curves recorded for each cycle. Each experimental group was set with at least 5 parallel samples.

### Rheological of Injectable PEGS/CPC Bone Cement

The rheological properties of bone cement at different temperatures were measured using a rotational rheometer (Thermo Scientific HAAKE MARS III, US) to investigate the factors influencing the curing time of the bone cement. The pre‐prepared paste was injected between two parallel plates (P20 TiLS, 20 mm in diameter), and then the upper plate was lowered until it just touched the paste. The experimental temperature was set at 25 °C or 37 °C, with constant strain of 1% and frequency of 10 Hz, and then record the variations in storage modulus (G′) and loss modulus (G″) over time. The time corresponding to the intersection point of the storage modulus (G′) and loss modulus (G″) curves was identified as the curing time.

### Hydration of Injectable PEGS/CPC Bone Cement

Investigating the impact of varying hydration durations on the mechanical properties of bone cement through compression testing. To mimic the hydration process of bone cement in vitro, no additional water was introduced during the injection of samples. The cured bone cement was immersed in water and then placed in a constant temperature chamber at 37 °C for 3, 6, 12, 24, and 48 h. The hydrated samples were subjected to compression testing, and stress‐strain curves were recorded to compare the changes in mechanical performance. The crystallinity and phase composition of CPC, L‐PEGS/CPC, and B‐PEGS/CPC were analyzed and characterized using X‐ray diffractometry (XRD). The sample was first ground into powder using an agate mortar and then analyzed using an XRD instrument. The data obtained was analyzed for crystallinity and crystal phase using XRDJADE software. In addition, XPS experiments were conducted on samples hydrated at different hydration times, and bond energy changes were analyzed using Avantage software.

### Hydration Heat Testing of L‐PEGS/CPC Bone Cement

Calorimetric measurements of the different paste compositions were performed at a TAM Air isothermal calorimeter (TA Instruments) with integrated thermostat (temperature variance ± 0.02 °C). It was equipped with eight twin type channels consisting of a sample and a reference chamber. Measurements were conducted by internal stirring using an InMixEr (Injection & mixing device for internal paste preparation, FAU Erlangen, Mineralogy). Here stirring of the samples was performed directly in the measurement chamber, which avoids influence of the initial heat flow by variations in the room temperature or disturbances due to opening of the measurement chambers. Hence, reliable data could be obtained even for the initial part of the cement hydration.

The sample was stirred at a constant speed of 1000 rpm for 1 min using an external motor. The temperature of the calorimeter was adjusted at 37 °C. Equilibration of the samples prior to measurement was performed directly in the measurement channel. The base line was observed to check when equilibration was completed. Three independent measurements of each sample were performed to check reproducibility. The heat flow curves were corrected for the calibration constant of the InMixEr tools and the time constant. Total heat release was determined by integration of the calorimetry curves.

### Porosity of Injectable PEGS/CPC Bone Cement

To determine the porosity of the material, bone cement slurry was filled into cylindrical molds (diameter: 6 mm, height: 10 mm). The cured samples were using Mercury intrusion porosimetry (Autopore V 9620) to determine the material's porosity. The pore size analysis range for the material was set at 5 nm to 800 µm.

### In Vivo Exothermic Behavior of PEGS/CPC Bone Cement

To investigate the in vivo exothermic behavior of bone cement, thermal images of the samples were captured at different time intervals using Infrared Thermal Camera (FOTRIC 220s), and the images were analyzed using FOTRIC AnalyzeIR software. Following anesthesia of SD rats, the mixed bone cement slurry was injected subcutaneously into the femur region. The curing process within the bone cement body was then recorded using an infrared thermal imaging camera.

### Degradation of PEGS/CPC Bone Cement

The in vivo degradation characteristics were investigated using an 8‐week‐old Sprague‐Dawley rat model. Cylindrical implants (3 mm diameter × 1 mm height) were fabricated in a mold, precisely weighed, and subsequently surgically introduced into the animals. Following explantation at predetermined intervals (7, 14, 21, and 28 days), the samples were dehydrated and mass measurements were recorded to plot the degradation kinetics. Weight variation is calculated according to the following formula:

(1) $$\mathrm{Weight}=\frac{M_{t}}{M_{0}}\times 100\%$$

where M_0_ denotes the dehydrated weight of the initial sample, and M_t_ denotes the dehydrated weight at each extraction time point.

### Cell Proliferation and Adhesion of Injectable PEGS/CPC Bone Cement

The cytotoxicity of bone cement was assessed according to the guidelines outlined in *GB/T 16 886.5‐2017*, with organotin‐containing PU used as the positive control group and HDPE as the negative control group. The soaking process was performed under sterile conditions using Dulbecco's Modified Eagle Medium (DMEM, Gibco) medium supplemented with 10% fetal bovine serum (FBS, Gibco), 1.0 × 10^5^ U L^−1^ penicillin (HyClone), and 100 mg L^−1^ streptomycin (HyClone) as the extraction medium. The bone cement samples were first weighed and then subjected to sterilization by UV irradiation. The sterile samples were immersed in an extraction solution with concentration of 0.2 g ml^−1^, and then placed in a 5% CO_2_ incubator at 37 °C for 24 h. Rat fibroblast (L929) cells were seeded into 96‐well plates at a density of 10000 cells per well and then placed in a 5% CO_2_ incubator at 37 °C for 24 h. Later, the extraction medium of samples was co‐cultured with L929 cells for 24 h. Under subdued light conditions, the extraction medium was removed, and 50 µL of thiazolyl blue (MTT, 1 mg mL^−1^) solution was added to each well. The 96‐well plate was incubated in a 5% CO_2_ incubator at 37 °C for 2 h, followed by the addition of 100 µL of dimethyl sulfoxide (DMSO) to each well. The plate was oscillated to ensure thorough dissolution of the formazan. The absorbance of the solution at a wavelength of 570 nm was measured using microplate reader (Spectra Max M2e, Molecular Devices, USA). Cell viability was calculated according to the following formula:

(2) $$\mathrm{Cell}\;\mathrm{viability}=\frac{OD_{570b}-OD_{570c}}{OD_{570b}}\times 100\%$$

where *OD*_570*c*_ is the mean absorbance value of the experimental group and *OD*_570*b*_ is the mean absorbance value of the blank group. When the cell viability was below 70% of the blank group, the sample was considered to have potential cytotoxicity. Upon confirming the L929 cytotoxicity of the material met national standard requirements, further cytotoxicity assays were conducted using BMSCs.

In the cell adhesion assay, rat bone marrow mesenchymal stem cells (rBMSCs) were first seeded onto the bone cement and cultured for 24 h. Subsequently, the cells were fixed with 2.5% glutaraldehyde solution for 15 min and washed three times with Phosphate buffer solution (PBS), for 10 min each. Glutaraldehyde fixation ensures superior cellular retention on the material scaffold, effectively preventing cell detachment during subsequent processing steps. Then the fixed cells were incubated with fluorescein isothiocyanate‐phalloidin (5 mg ml^−1^, Sigma‐Aldrich), 4′,6‐diamidino‐2‐phenylindole (DAPI, 5 mg ml^−1^, Sigma‐Aldrich) and 1% Sudan black B for cytoskeleton, cellular nuclei staining and shielding the auto‐fluorescent of polymer, respectively. The samples were fluorescently observed and images were captured using laser scanning confocal microscopy (LSCM, Nikon A1R, Japan).

The cytocompatibility and proliferative effects of the materials were assessed using rBMSCs. Cells were exposed to extracts of the materials, with experimental groups (varying extract of materials), a negative control (cells in complete medium), and a blank (cell‐free medium) established. Proliferation was quantitatively evaluated using the CCK‐8 method. Cell proliferation was calculated according to the following formula:

(3) $$\mathrm{Cell}\;\mathrm{proliferation}=\frac{OD_{450c}-OD_{450a}}{OD_{450b}-OD_{450a}}\times 100\%$$

where *OD*_450*c*_ is the mean absorbance value of the experimental group at 450 nm, *OD*_450*b*_ is the mean absorbance value of the negative control group, and *OD*_450*a*_ is the mean absorbance value of the blank group.

### Osteogenic Differentiation of Injectable PEGS/CPC Bone Cement

The osteogenic activity of bone cement was evaluated via staining of ALP and calcium nodule using ALP staining kit (Beyotime, Wuhan, China) and Alizarin red staining kit (Cyagen Bioscience, China). The rBMSCs were seeded in 24‐well plates at a density of 20000 cells per well and co‐cultured with the material extracts. The sterile materials were positioned in Transwell inserts and immersed in culture medium. The medium was refreshed every two days. After 7 and 14 days of culture, the rBMSCs were stained with ALP to assess osteogenic differentiation level. Additionally, the ALP activity was measured using an ALP activity assay kit (Beyotime, China). After 14 or 21 days, the number of calcium nodules was analyzed by ARS staining.

### RNA Analysis Using qRT‐PCR

Total RNA extracts were obtained through the lysis of BMSCs with Trizol (TAKARA, Japan) after three days and seven days of treatment following the manufacturer's instructions. The purity and concentration were quantified using NanoDrop 2000 (Invitrogen, USA). Reverse transcription was performed using PrimeScript RT reagent kit (TAKARA, RR037A) following the manufacturer's instructions. PCRs were performed using TB Green Premix Ex Taq kit (TAKARA, RR420A), following the manufacturer's instructions. The primer sequences used are listed in Table S2 (Supporting Information).

### Regeneration of Rat Femoral Defects

All animal experiments were approved by the Animal Research Committee of Shanghai Jiao Tong University School of Medicine (Shanghai, China), the Animal Ethics number was 20 180 901. The animal experimental procedures adhered to the Animal Research: Reporting of in Vivo Experiments (ARRIVE) guidelines and the Guide for the Care and Use of Laboratory Animals by the National Institutes of Health (NIH publication No. 8023, revised 1978). 12‐week‐old SD rats weighing ≈500 g were selected to establish the rat femoral defect model. Anesthesia was induced in rats by intraperitoneal injection of sodium pentobarbital solution. The fur around the femoral area was shaved and sterilized with alcohol. The skin was incised using a sterile surgical blade to expose the lateral aspect of the femur. A circular defect with a diameter of 3 mm was created on the lateral aspect of the femur using an electric trephine drill, followed by injection of the material into the defect (CSD model: the smallest osseous defect that will not spontaneously heal over an organism's lifetime). The experimental groups were arranged as follows: 1) Blank control; 2) PMMA bone cement; 3) L‐PEGS/CPC bone cement. Each experimental group was set with four parallel samples. Finally, the wound was sutured, and after 12 weeks of normal feeding, tissue specimens of the femur were obtained.

### Micro‐Computed Tomography (micro‐CT) Measurements and Evaluation

The experimental rats euthanized with an overdose of anesthesia after 12 weeks post‐operation, and the femurs were then extracted and immersed in 4% paraformaldehyde. The specimens were subjected to 3D rotational scanning analysis using micro‐CT technology. Volume and morphometric analyses were performed using Scanco Medical AG (Switzerland) software, including measurements of parameters such as bone volume fraction (bone volume/tissue volume, BV/TV, %), trabecular number (Tb.N, mm^−1^), Trabecular Separation/Spacing (Tb.sp, mm) and so on.

### Histological Analysis

8‐week‐old, ≈300 g Sprague‐Dawley (SD) rats were selected for in vivo safety assessment. After anesthetizing the rats to a state of muscle relaxation, a small incision was made at the muscle dorsal skin using sterile surgical instruments, followed by implantation of the material. Two weeks after feeding, rats were euthanized under anesthesia, and the implants along with surrounding tissues were excised and fixed in 4% paraformaldehyde solution for subsequent histological analysis. The fixed specimens need decalcification of hard tissues. The samples were immersed in a 12.5% EDTA solution and kept at 37 °C for one month. The samples were dehydrated using a gradient concentration of ethanol, followed by embedding in paraffin wax, and finally sectioned using a microtome (Leica 1600, Germany). Histological sections were stained with Hematoxylin and Eosin (H&E) stain to observe the tissue compatibility of the materials.

In bone defect repair experiments, femoral specimens were harvested and fixed in 4% paraformaldehyde. For the PMMA group, the cement was surgically extracted prior to sectioning. Given its rigid and high‐strength nature, PMMA tends to induce delamination and fragmentation of adjacent bone tissue during microtome sectioning, thereby compromising subsequent histological analysis. Owing to the bioinert nature of PMMA, which precludes chemical bonding with bone tissue, the material could be completely removed using surgical instruments, revealing an intact osseous bed. All samples subsequently underwent dehydration, embedding, and microtome sectioning. Tissue sections were subjected to Van Gieson (VG) staining to evaluate bone repair through visualization of bone matrix and collagen deposition.

### Statistical Analysis

All experimental data were generated from at least three independent experiments, and the results were presented as the mean ± standard deviation (SD). All analyses were performed using one‐way analysis of variance (ANOVA). The significance differences were accepted at *p < 0.05, **p < 0.01, ***p < 0.001, and ****p < 0.0001.

## Conflict of Interest

The authors declare no conflict of interest.

## Supporting information



Supporting Information

Supplemental Video 1

Supplemental Video 2

## Data Availability

The data that support the findings of this study are available in the supplementary material of this article.
