# Supplementary material for: Injectable and In Situ Hydration‐Reinforced Hybrid Bone Cements for Accelerated Bone Regeneration
Source: Adv Sci (Weinh). 2025 Nov 16;13(7):e12723. doi: 10.1002/advs.202512723 (PMC12866785; doi:10.1002/advs.202512723)
Supplement: Supplementary file 1 — Supporting Information [file ADVS-13-e12723-s001.docx]

Supporting information

**Injectable and In Situ Hydration-Reinforced Hybrid Bone Cements for Accelerated Bone Regeneration**

*Xing Chen ^a, b, #^, Yifan Ma ^c, #^, Lingfei Zhao ^a, b^, Lingbin Che ^d^, Dianwen Song ^d^, Zihan Wu ^e,^ *, Changsheng Liu ^a, b,^ *, Yuan Yuan ^a, b,^ **

^a^ Key Laboratory for Ultrafine Materials of Ministry of Education, School of Materials Science and Engineering, East China University of Science and Technology, Shanghai, 200237, P.R. China

^b^ Engineering Research Center for Biomedical Materials of the Ministry of Education, East China University of Science and Technology, Shanghai, 200237, P.R. China

^c^ Department of Radiation Oncology, The University of Texas MD Anderson Cancer Center, Houston, TX, USA

^d^ Department of Orthopedics, Shanghai General Hospital, Shanghai Jiaotong University School of Medicine, Shanghai, 200080, P.R. China.

^e^ Shanghai Rebone Biomaterials Co., Ltd, Shanghai 201707, P.R. China.

# These authors contributed equally.

* Corresponding author.

E-mail address: wuzihan@rebone.com (Z. Wu), liucs@ecust.edu.cn (C. Liu), yyuan@ecust.edu.cn (Y. Yuan).

**Tables and Figures**

**Table S1: GPC, thermodynamic parameters and hydroxyl content of B-PEGS and L-PEGS**

| **Polymer** | ***Mw* (kDa)** | **PDI** | **T_g_(℃)** | **T_c_(℃)** | **T_m_(℃)** | **Hydroxyl Value (*HV*)** |
| --- | --- | --- | --- | --- | --- | --- |
| **B-PEGS** | 12.0 | 3.74 | -51.3 | -39.5 | 22.4 | 9.48 |
| **L-PEGS** | 10.6 | 1.30 | N/A | N/A | N/A | 39.87 |

**Table S2: PCR primer sequences**

| **Gene** | **Name** | **Sequence (5'-3’)** |
| --- | --- | --- |
| **ALP** | Forward | TGGAACACTGGGTCCCATA |
|  | Reverse | GACCTGGTCTTCCCTCCAA |
| **COL-1** | Forward | TGGATGGCTGCACGAGT |
|  | Reverse | TTGGGATGGAGGGAGTTTA |
| **RUNX2** | Forward | ATCCAGCCACCTTCACTTACACC |
|  | Reverse | GGGACCATTGGGAACTGATAGG |
| **OCN** | Forward | GCCCTGACTGCATTCTGCCTCT |
|  | Reverse | TCACCACCTTACTGCCCTCCTG |


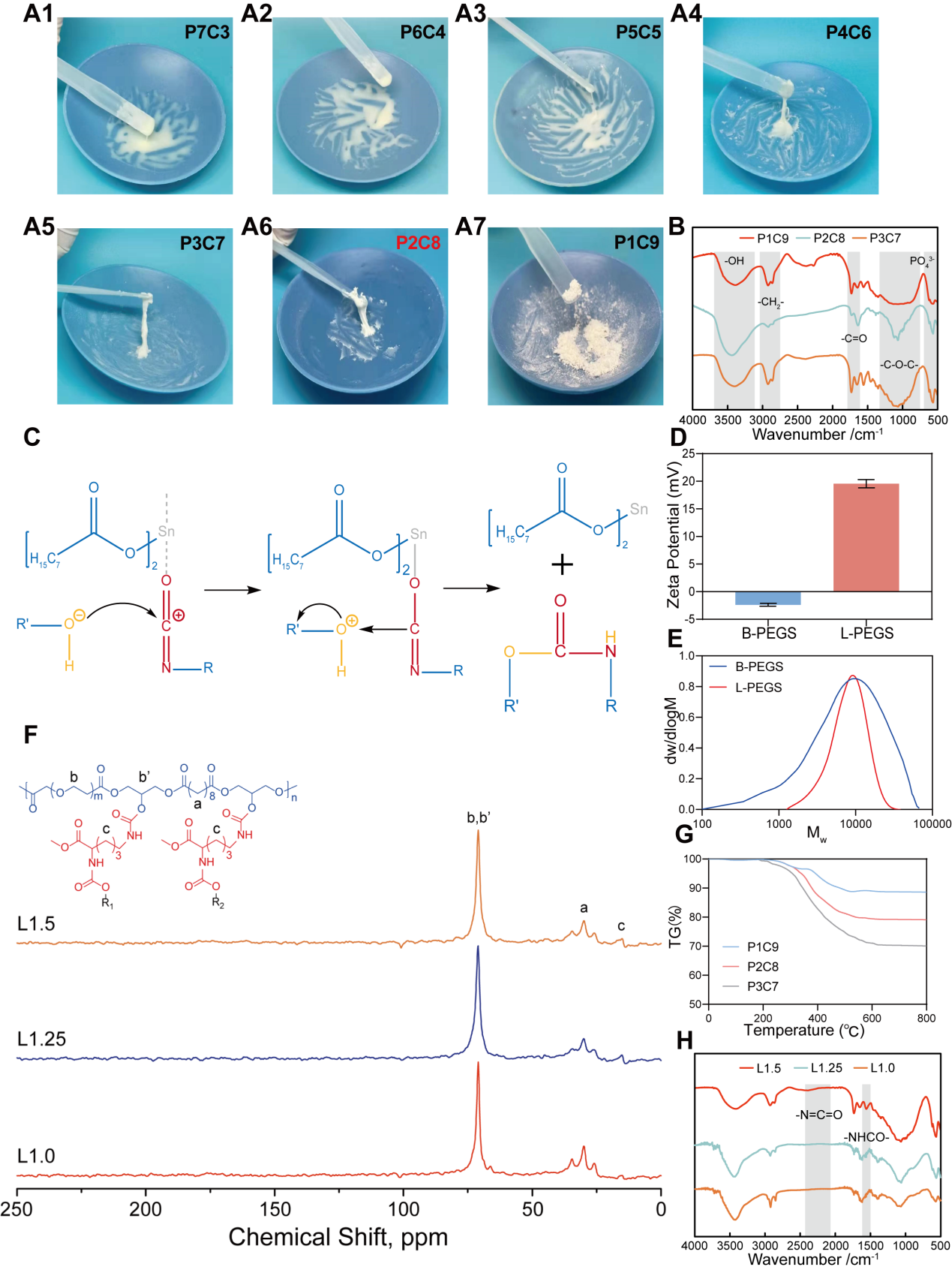


**Figure S1.** (A) State of bone cement slurries with different L-PEGS/CPC composite ratios (PxCy, Mass ratio of L-PEGS to CPC in bone cement is x: y). (B) FT-IR spectrum of bone cement with various L-PEGS/CPC composite proportion in the range of 500-4000 cm^-1^. (C) The catalytic mechanism of stannous octanoate. (D) The zeta potential of B-PEGS and L-PEGS. (E) The GPC curve indicates that the molecular weight distribution of linear PEGS synthesized by acid induced epoxy ring opening method is narrower. (F) ^13^C NMR spectra of L-PEGS/CPC with various LDI amount. (G) TG curves of bone cement with various L-PEGS/CPC composite proportion. (H) FT-IR spectrum of L-PEGS/CPC with various LDI amount (Lz, z represents the ratio of isocyanate groups to hydroxyl groups in the bone cement system). No signal was detected at 2250 cm^-1^, indicating that the isocyanate in the bone cement had fully reacted.

**
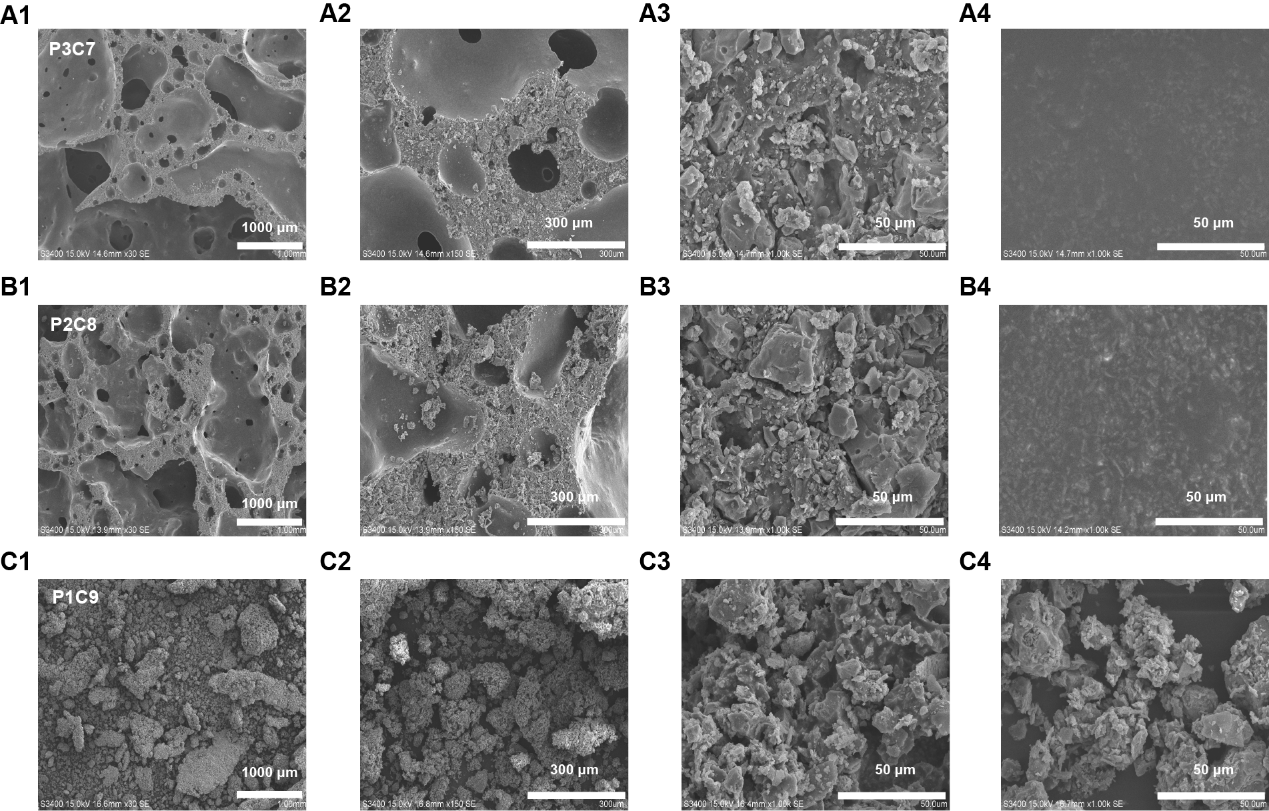
**

**Figure S2.** SEM images of (A) P3C7, (B) P2C8 and (C) P1C9 at different magnifications. Scale bar: 1000μm, 500μm, 50μm and 50μm respectively.

***
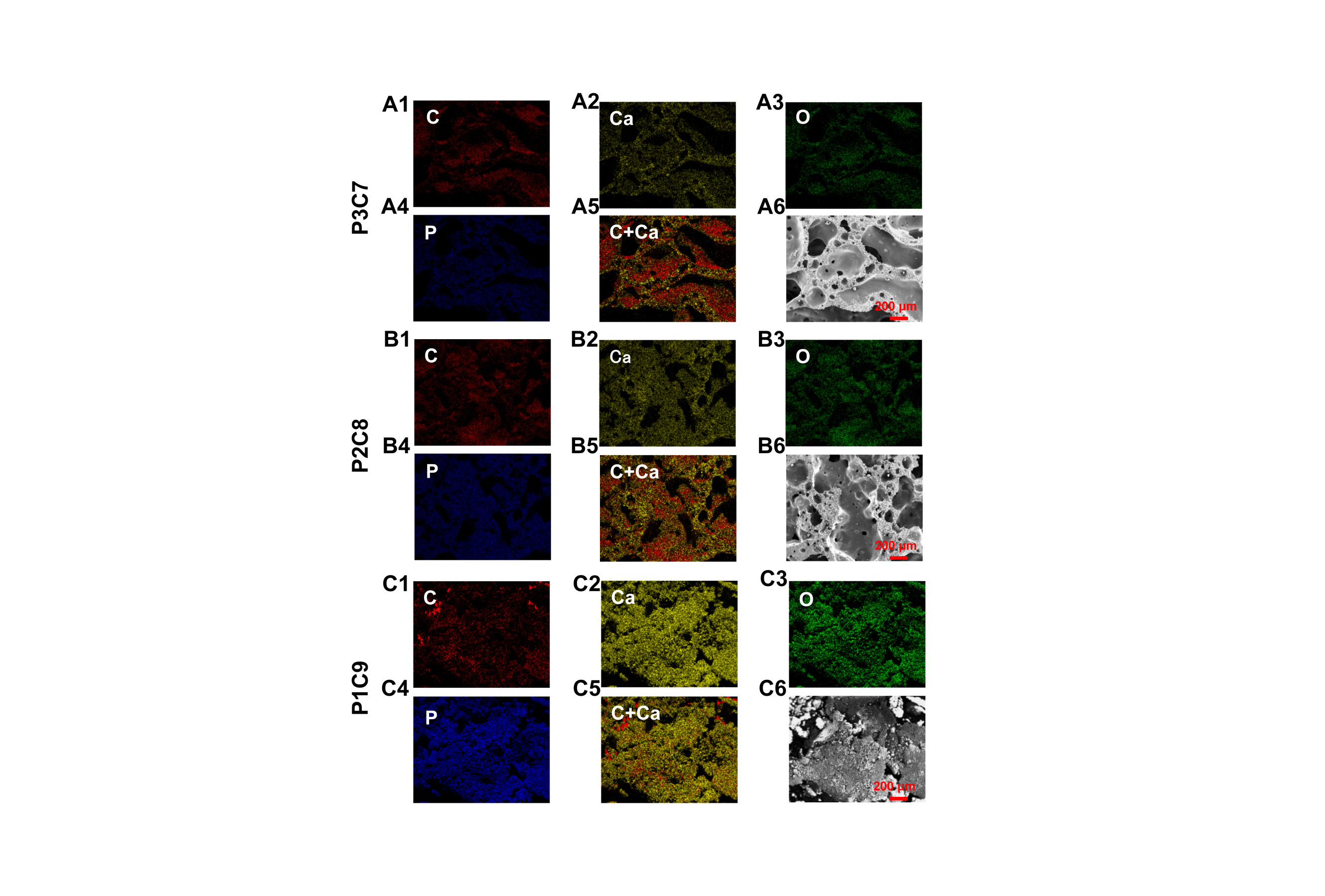
***

**Figure S3.** Surface Element Analysis of (A) P3C7, (B) P2C8 and (C) P1C9. Scale bar: 200μm.


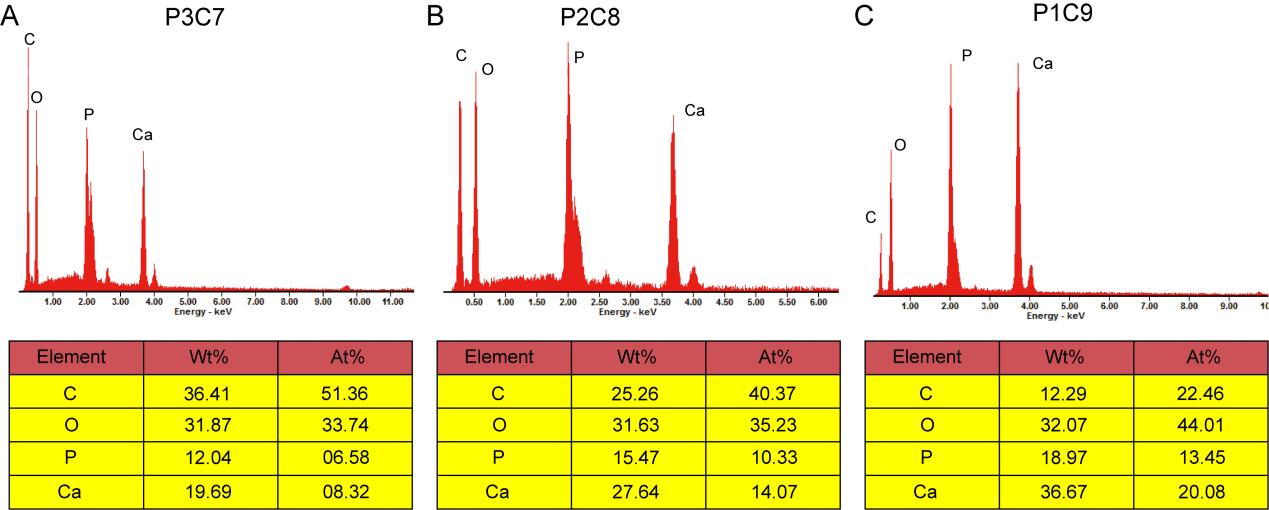


**Figure S4.** Elements analysis of (A) P3C7, (B) P2C8 and (C) P1C9.


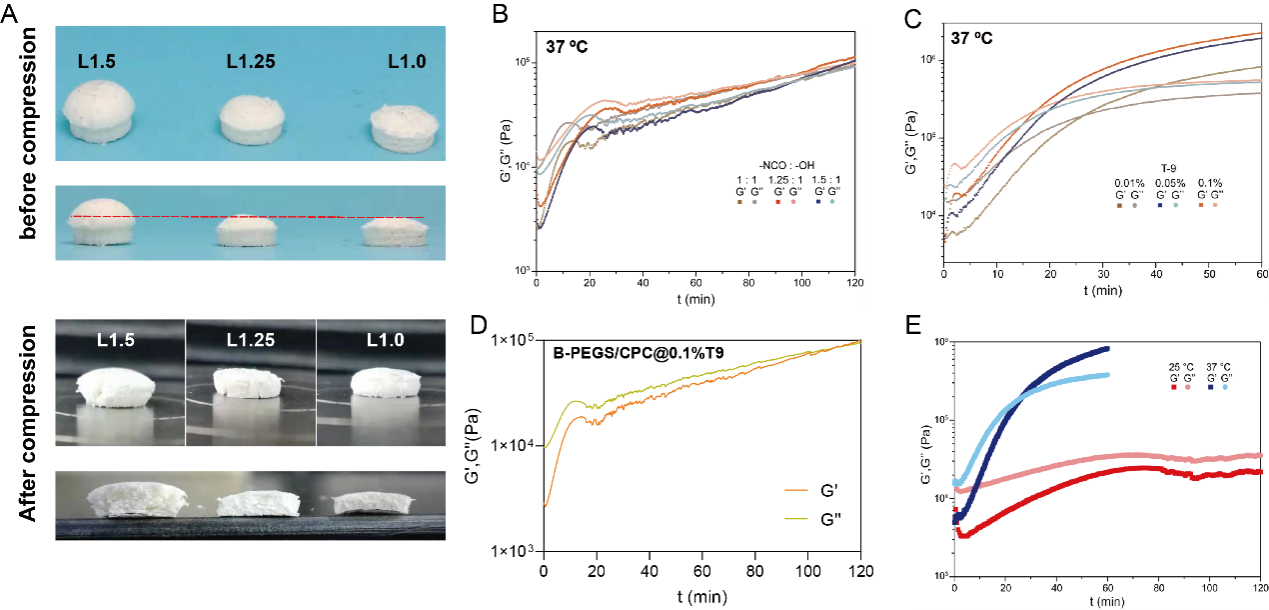


**Figure S5.** (A) Photographs of compressed L-PEGS/CPC bone cement with various LDI amount. (B) Rheological analysis of L-PEGS/CPC without stannous octanoate. (C) Rheological analysis of L-PEGS/CPC with various stannous octanoate amount. (D) Rheological analysis of B-PEGS/CPC with 0.1% stannous octanoate. (E) Rheological analysis of L-PEGS/CPC at room temperature and physiological temperature. L-PEGS/CPC exhibits excellent injectability and operability, which could achieve solidificaction within 30 minutes in vivo and maintain injectability for more than 120 minutes in vitro.


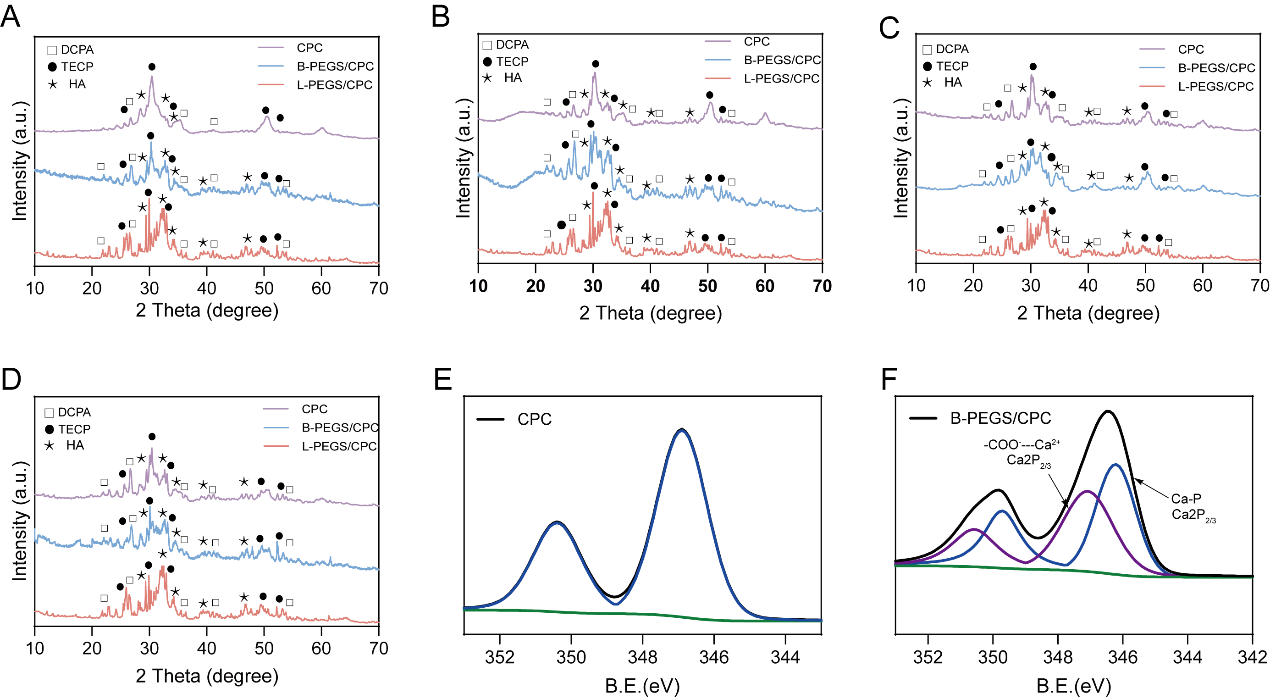


**Figure S6.** The XRD pattern of bone cement after (A) 3 hours, (B) 6 hours, (C) 24 hours and (D) 48 hours of hydration (The peak of DCPA, TECP and HA are indicated with □, ● and ★). (E) XPS spectra of Ca 2p_3/2_ and 2p_1/2_ of CPC. (F) XPS spectra of Ca 2p_3/2_ and 2p_1/2_ of B-PEGS/CPC.


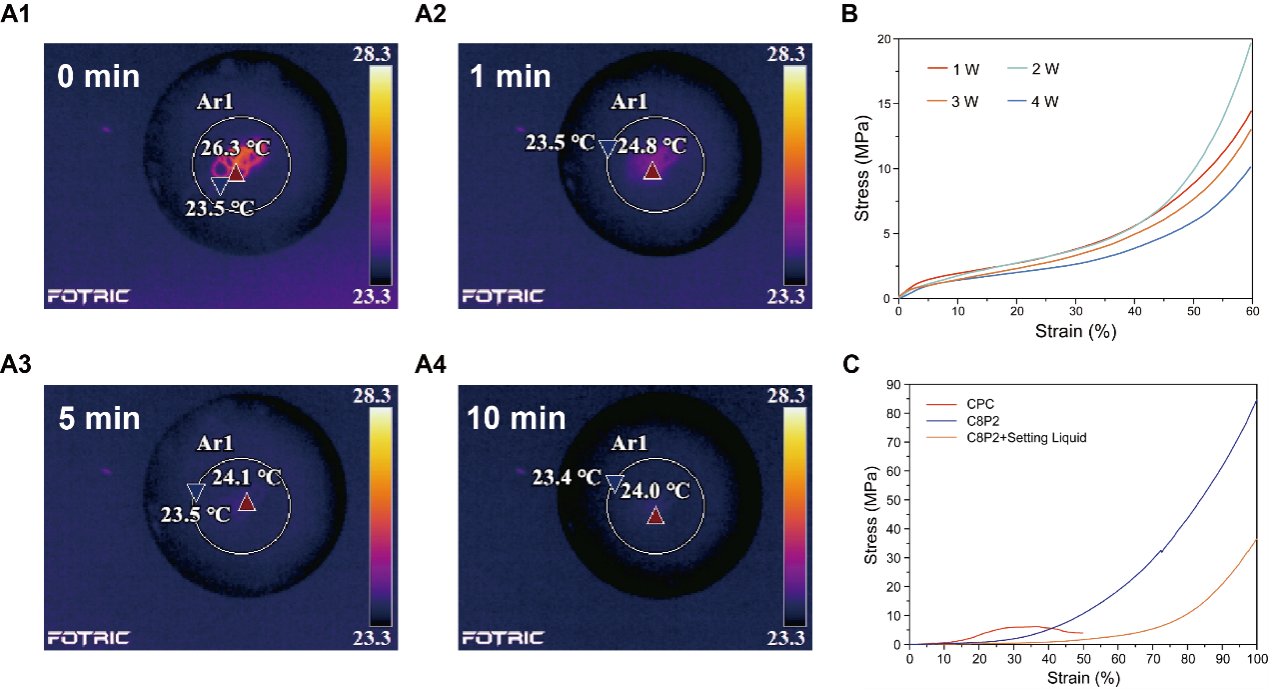


**Figure S7.** (A) Infrared thermal images of L-PEGS/CPC after injection at different time points. (B) The mechanical strength of L-PEGS/CPC bone cement after different degradation times. (C) The mechanical strength of bone cement before and after hydration.


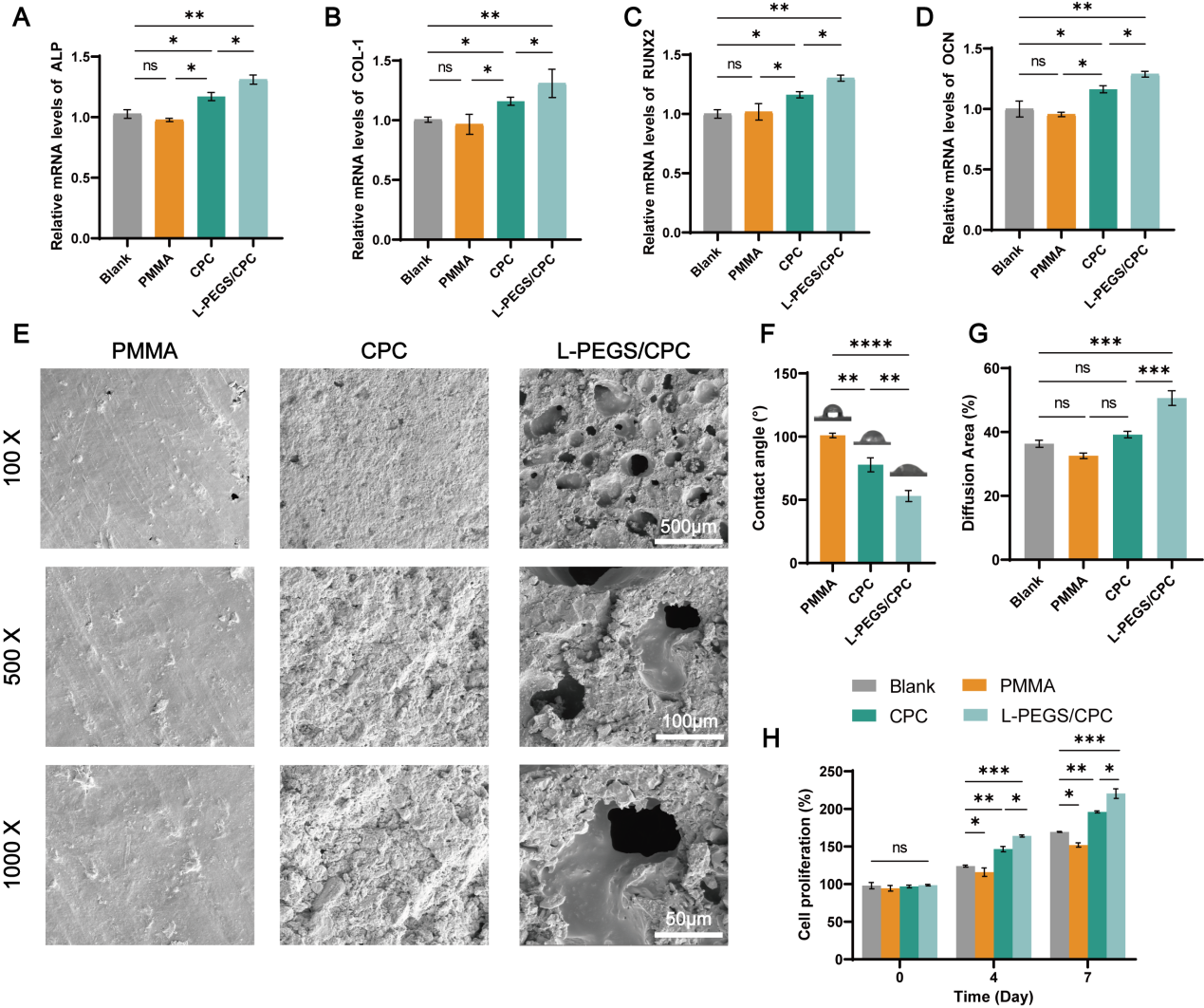


**Figure S8.** qRT-PCR analysis of (A) ALP, (B) COL-1, (C) RUNX2 and (D) OCN in BMSCs with the treatment of CPC, L-PEGS/CPC and PMMA for 3 days. (E) Surface architecture of L-PEGS/CPC, CPC and PMMA. The surface of L-PEGS/CPC has a porous structure that facilitates cell growth and adhesion. (F) Contact angle of L-PEGS/CPC, CPC and PMMA. L-PEGS/CPC exhibits excellent surface hydrophilic properties. (G) The diffusion area of BMSCs on PMMA, CPC, and L-PEGS/CPC. (H) Proliferative activity of BMSCs in L-PEGS/CPC, CPC and PMMA, as assessed by the CCK-8 method, after four and seven days of culture. (mean ± SD; n = 5; *p* ≥0.05 (no significant, ns), **p* < 0.05; ***p* < 0.01; ****p* < 0.001, *****p* < 0.0001).


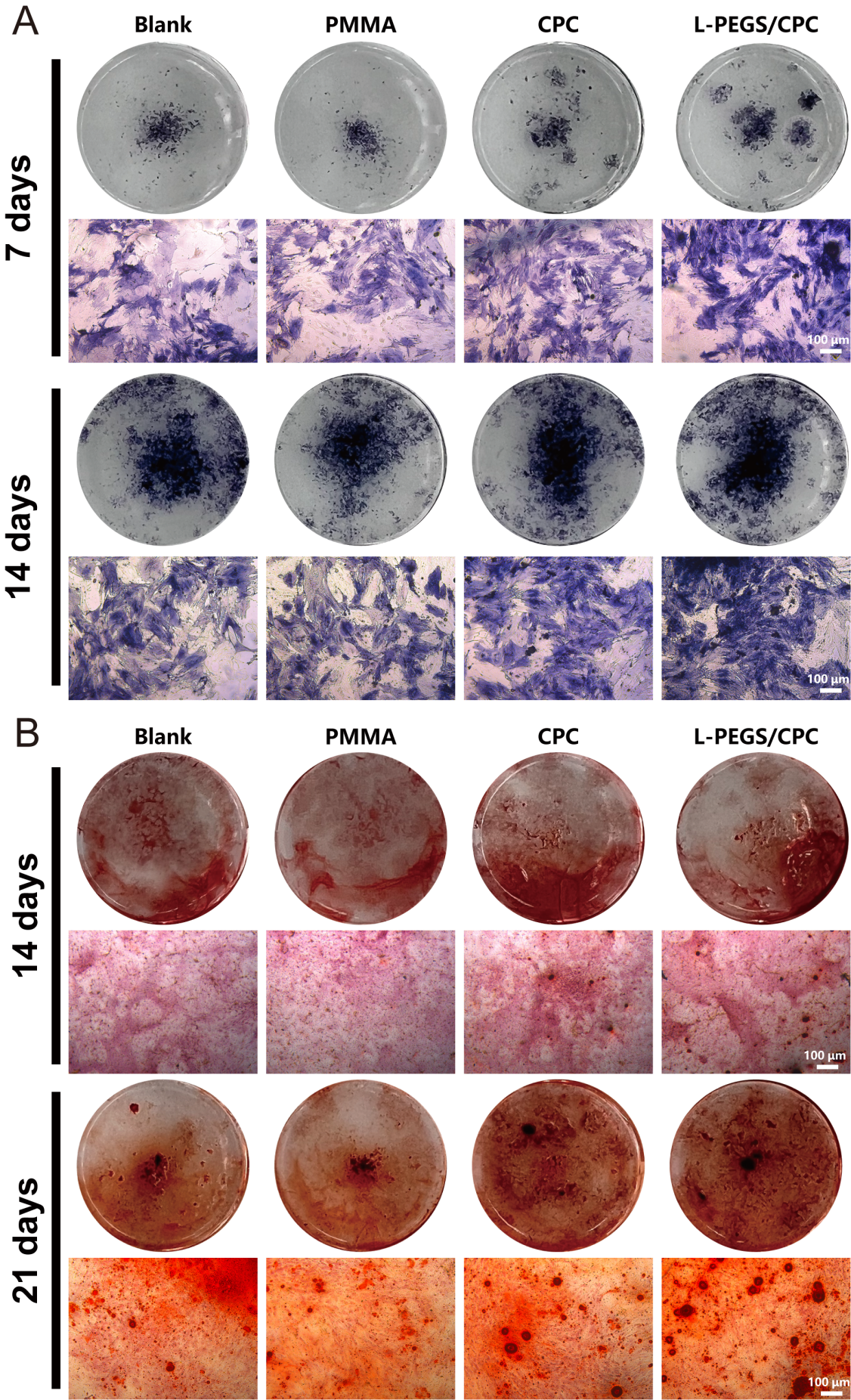


**Figure S9.** (A) ALP staining of BMSCs co-cultured with CPC, L-PEGS/CPC and PMMA for 7 days and 14 days. (Scale bar:100 μm). (B) ARS staining of BMSCs co-cultured with CPC, L-PEGS/CPC and PMMA for 14 days and 21 days. (Scale bar:100 μm).


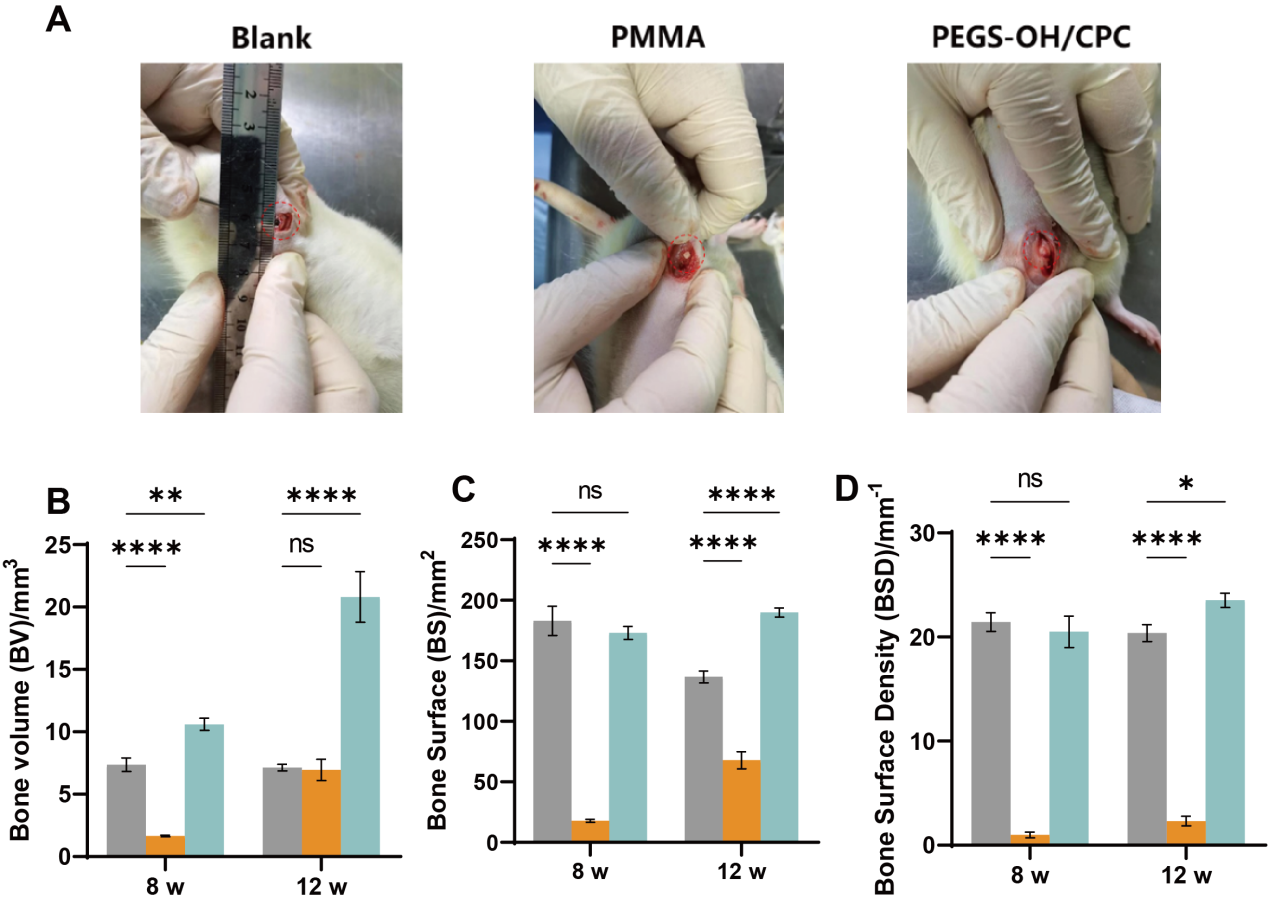


**Figure S10.** (A) Establishment of the femoral defect model in SD rats. Quantification of (B) bone volume (BV, mm^3^), (C) bone surface (BS, mm^2^) and (D) bone surface density (BSD, mm^-1^) derived from micro-CT at 8 weeks and 12 weeks. (mean ± SD; n = 5; *p* ≥0.05 (no significant, ns), **p* < 0.05; ***p* < 0.01; ****p* < 0.001, *****p* < 0.0001)


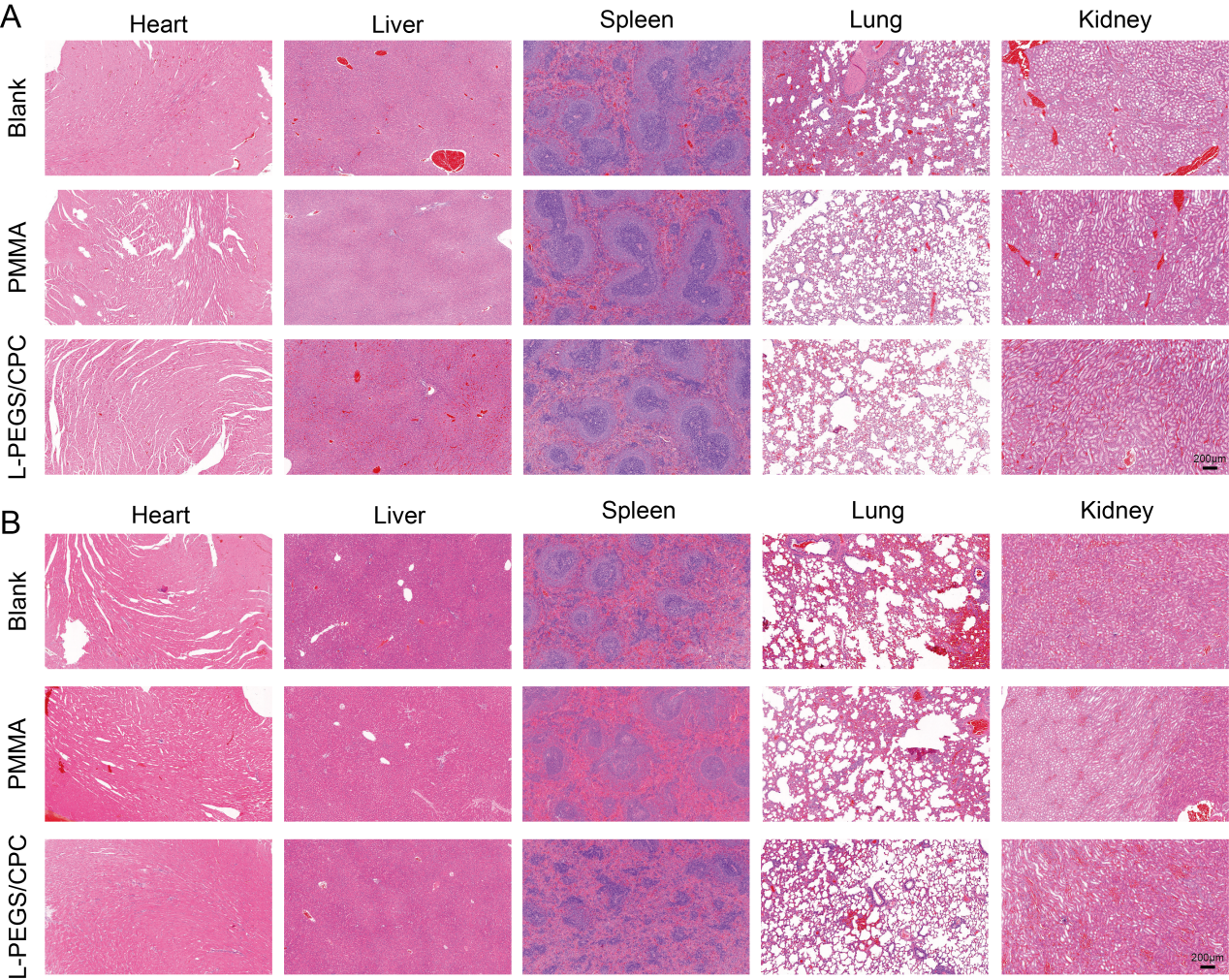


**Figure S11.** (A) Histological and pathological evaluation of major organs in rats treated with bone cement at 8 weeks. (B) Histological and pathological evaluation of major organs in rats treated with bone cement at 12 weeks.
